# Nanoscale metal-organic frameworks for mitochondria-targeted radiotherapy-radiodynamic therapy

**DOI:** 10.1038/s41467-018-06655-7

**Published:** 2018-10-17

**Authors:** Kaiyuan Ni, Guangxu Lan, Samuel S. Veroneau, Xiaopin Duan, Yang Song, Wenbin Lin

**Affiliations:** 1 Department of Chemistry, The University of Chicago, Chicago, IL 60637 USA; 2 Department of Radiation and Cellular Oncology, Ludwig Center for Metastasis Research, The University of Chicago, Chicago, IL 60637 USA

## Abstract

Selective delivery of photosensitizers to mitochondria of cancer cells can enhance the efficacy of photodynamic therapy (PDT). Though cationic Ru-based photosensitizers accumulate in mitochondria, they require excitation with less penetrating short-wavelength photons, limiting their application in PDT. We recently discovered X-ray based cancer therapy by nanoscale metal–organic frameworks (nMOFs) via enhancing radiotherapy (RT) and enabling radiodynamic therapy (RDT). Herein we report Hf-DBB-Ru as a mitochondria-targeted nMOF for RT-RDT. Constructed from Ru-based photosensitizers, the cationic framework exhibits strong mitochondria-targeting property. Upon X-ray irradiation, Hf-DBB-Ru efficiently generates hydroxyl radicals from the Hf_6_ SBUs and singlet oxygen from the DBB-Ru photosensitizers to lead to RT-RDT effects. Mitochondria-targeted RT-RDT depolarizes the mitochondrial membrane to initiate apoptosis of cancer cells, leading to significant regression of colorectal tumors in mouse models. Our work establishes an effective strategy to selectively target mitochondria with cationic nMOFs for enhanced cancer therapy via RT-RDT with low doses of deeply penetrating X-rays.

## Abstract (web summary)

Photosensitizers delivered to mitochondria of cancer cells can enhance photodynamic therapy. Here, the authors report mitochondria-targeted radiation therapy and radiodynamic therapy of colorectal cancer models with a cationic nanoscale metal-organic framework to overcome shallow light penetration of Ru-based photosensitizers.

## Introduction

Photodynamic therapy (PDT) has provided an effective local cancer treatment by eradicating malignant tumors without damaging surrounding normal tissues^1–4^. In PDT, molecular oxygen is converted into highly cytotoxic reactive oxygen species (ROS), typically singlet oxygen (^1^O_2_), by photosensitizers (PSs) in their excited states^5^. Since ^1^O_2_ has a short lifetime in biological systems (~40 ns) and limited radius of diffusion from its site of generation (<30 nm)^6^, PDT selectively targets the locale of a PS at the time of light irradiation^7,8^. As a result, the therapeutic effect of PDT is greatly influenced by localization of PSs in specific subcellular organelles.

Several subcellular organelles, including mitochondria^9^, lysosomes^10^, and plasma membranes^11^, have been evaluated as potential PDT targets. In particular, mitochondria have been recognized as a novel pharmacological target for cancer treatment due to their central role in mediating cell apoptosis^12–14^. Mitochondria undertake critical functions in various biological processes of cells, including energy production, molecular metabolism, and redox status maintenance. Mitochondrial dysfunction can interrupt energy supply and activate mitochondria-mediated apoptotic pathways^15^. In cancer, mitochondria play key roles on tumor cell proliferation, invasion, and metastasis. Thus, generating ^1^O_2_ inside mitochondria can damage them at the early stage of PDT treatment to maximize the cytotoxic effect. Consequently, there is significant interest in developing mitochondria-targeted PSs to enhance PDT effects and improve cancer treatment.

Cationic ruthenium (Ru)-based PSs have recently been reported to target mitochondria^16–18^ without the need of conjugating extrinsic mitochondria-targeting moieties such as the triphenylphosphonium group^19,20^. Ru-based PSs possess several favorable properties including long excited state lifetimes, high ^1^O_2_ generation efficiencies, and good aqueous solubility^21^. Unfortunately, due to large Stoke shifts, Ru-based PSs can only be excited with light at short wavelengths, which has shallow tissue penetration depth (<0.1 cm)^22^. Although two-photon excitation can be used to activate Ru-based PSs for mitochondria-targeted PDT, the two-photon process has low photosensitizing efficiency^23–25^. Innovative strategies are thus needed to realize anti-cancer PDT treatment with mitochondria-targeted Ru-based PSs.

As a new class of molecular nanomaterials, nanoscale metal–organic frameworks (nMOFs) have shown interesting potential in nanomedicine applications due to their synthetic tunability, multifunctionality, and biocompatibility^26–29^. The ordered crystalline structures and intrinsic porosity of nMOFs not only avert self-quenching but also facilitate the diffusion of reactive oxygen species (ROSs) to improve the efficacy of PDT^30–34^. We recently discovered efficient X-ray based cancer therapy by Hf-porphyrin nMOFs via enhancing radiotherapy (RT) and enabling radiodynamic therapy (RDT) using low doses of deeply penetrating X-rays^35,36^. Upon X-ray irradiation, Hf-porphyrin nMOFs efficiently generated hydroxyl radicals from the Hf SBUs and singlet oxygen from the porphyrin PSs to exert RT-RDT effects.

Herein we report the synthesis of Hf-DBB-Ru [DBB-Ru = bis(2,2’-bipyridine)(5,5’-di(4-benzoato)-2,2’-bipyridine)ruthenium(II) chloride] as a mitochondria-targeted nMOF for RT-RDT. By integrating Ru(bpy)_3_^2+^ PSs into the framework, Hf-DBB-Ru possesses a cationic UiO topology with a formula of [Hf_6_(µ_3_-O)_4_(µ_3_-OH)_4_(DBB-Ru)_6_]^12+^ and exhibits strong mitochondria-targeting property as demonstrated by elemental quantification and super-resolution confocal microscopy. Upon irradiation with low doses of highly penetrating X-rays, Hf-DBB-Ru enables RT-RDT by efficiently generating hydroxyl radicals from the Hf_6_ SBUs and singlet oxygen from the DBB-Ru PSs. Mitochondria-targeted RT-RDT process depolarizes the mitochondrial membrane potential, releases cytochrome c, and disturbs the respiratory chain to lead to programmed cell death of tumor cells. We further show that nMOF-enabled mitochondria-targeted RT-RDT significantly regresses colorectal tumors on mouse models at very low X-ray doses and with no side effects.

## Results

### nMOF synthesis and characterization

**Fig. 1:**
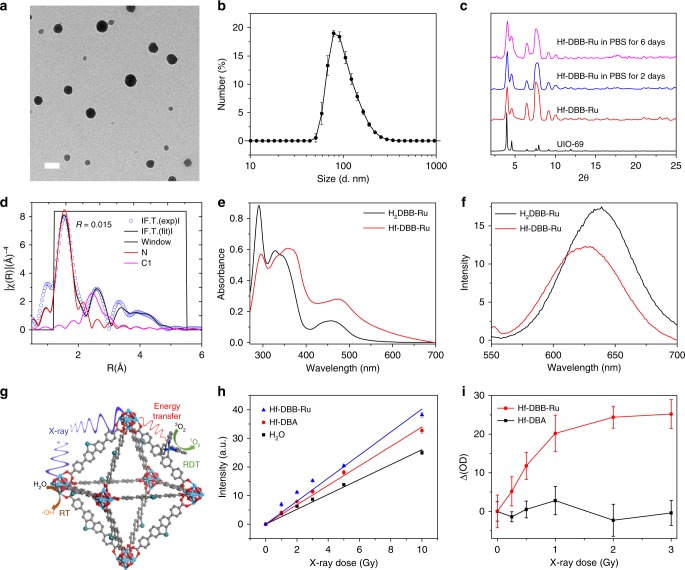
Characterization of Hf-DBB-Ru. **a** TEM image of Hf-DBB-Ru. **b** Number-averaged diameter of Hf-DBB-Ru in water by DLS measurements, *n* = 3. **c** PXRD patterns of Hf-DBB-Ru samples and UiO-69. Hf-DBB-Ru was freshly prepared or incubated in 0.6 mM PBS for 6 days. **d** EXAFS fitting of Hf-DBB-Ru. **e** UV-visible spectra of Hf-DBB-Ru and H_2_DBB-Ru. **f** Emission spectra of Hf-DBB-Ru and H_2_DBB-Ru with 450 nm excitation. **g** Schematic showing the RT and RDT process enabled by Hf-DBB-Ru. **h** APF fluorescence of Hf-DBB-Ru and Hf_6_-DBA at equivalent Hf concentrations of 20 µM and H_2_O upon X-ray irradiation, *n* = 6. **i** SOSG fluorescence of Hf-DBB-Ru and Hf_6_-DBA at equivalent Hf concentrations of 20 µM upon X-ray irradiation, *n* = 6. The error bars represent s.d. values. The TEM images were obtained with five repetitions to afford similar results

Hf-DBB-Ru was synthesized through a solvothermal reaction between HfCl_4_ and H_2_DBB-Ru with trifluoroacetic acid as the modulator (Supplementary Methods and Supplementary Fig. 1). Transmission electron microscopy (TEM) imaging showed a uniform spherical morphology of <100 nm in diameter for Hf-DBB-Ru (Fig. 1a), whereas dynamic light scattering (DLS) measurements gave a Z-average diameter, number-average diameter, and polydispersity index of 98.1 ± 4.1 nm, 76.0 ± 3.9 nm, and 0.12 ± 0.01, respectively, for Hf-DBB-Ru (Fig. 1b). Hf-DBB-Ru particles exhibited a positive ζ potential of 38.9 ± 3.1 mV due to the cationic [DBB-Ru]^2+^ bridging ligands. Powder X-ray diffraction (PXRD) studies showed that Hf-DBB-Ru adopted a UiO-like topological structure (Fig. 1c). Though Hf-DBB-Ru showed the same peak positions as the reported UiO-69, the first peak at 2*θ* = 3.96 °, which corresponds to the (111) reflection, exhibited lower intensity than UiO-69 (Fig. 1c), likely due to out-of-phase destructive interference from Ru centers which are sitting half way between the in-phase Hf clusters. Based on the UiO-69 topological structure, the cationic Hf-DBB-Ru framework was formulated as [Hf_6_(µ_3_-O)_4_(µ_3_-OH)_4_(DBB-Ru)_6_]^12+^. The Ru K-edge features of extended X-ray absorption fine structure (EXAFS) of Hf-DBB-Ru were well fitted with the structure model of Ru(bpy)_3_^2+^ (Fig. 1d and Supplementary Fig. 2, Supplementary Methods, Supplementary Tables 1, 2), indicating their similar Ru coordination environments. Hf-DBB-Ru showed similar absorption peaks and phosphorescence peak to the H_2_DBB-Ru ligand (Fig. 1e, f). Finally, PXRD studies showed that Hf-DBB-Ru was stable in 0.6 mM PBS for 6 days owing to strong carboxylate coordination to the Hf_6_ clusters (Fig. 1c). Hf-DBA, a neutral MOF with a ζ potential of −24.1 ± 5.6 mV in water, was synthesized as previously reported^37^. Hf-DBA exhibited similar size and morphology as Hf-DBB-Ru (Supplementary Fig. 3).

### Hydroxyl radical and singlet oxygen generation

We next determined if Hf-DBB-Ru could generate both hydroxyl radical and singlet oxygen upon X-ray irradiation (Fig. 1g). Hydroxyl radicals were detected via aminophenylfluorescein (APF) assay, in which APF reacts with hydroxyl radicals to give bright green fluorescence at 515 nm upon excitation at 490 nm. The amount of APF trapped in Hf-DBB-Ru or Hf-DBA was first estimated by detecting chemically produced hydroxyl radicals using APF in the presence of the nMOF. The percentage of APF trapped in the nMOF is approximated to one minus the fluorescence intensity ratio of APF with nMOF over that of APF without nMOF (Supplementary Methods and Supplementary Fig. 4). An aqueous solution or an aqueous dispersion of Hf-DBB-Ru or Hf-DBA at a Hf concentration of 20 μM was added 5 μM APF and then irradiated with X-ray in the dose range of 0 to 10 Gy. The fluorescence signals at 515 nm were detected. We deduced the fluorescence intensity corresponding to the amounts of hydroxyl radicals generated by Hf-DBB-Ru, Hf-DBA, and H_2_O by correcting for the percentage of APF trapped in the nMOF (Fig. 1h). Hydroxyl radical generation for all groups increased linearly with X-ray dose and the relative enhancement of hydroxyl radical generation compared with water was determined to be 54.2% and 30.5% for Hf-DBB-Ru and Hf-DBA, respectively.

We used singlet oxygen sensor green (SOSG) assay to detect singlet oxygen generation by Hf-DBB-Ru. 12.5 μM SOSG was added to an aqueous solution or an aqueous dispersion of Hf-DBB-Ru or Hf-DBA at a Hf concentration of 20 μM and irradiated with X-ray in the dose range of 0 to 3 Gy. The fluorescence signals at 540 nm for DBB-Ru and Hf-DBA were determined by subtracting that of an aqueous solution. As shown in Fig. 1i, Hf-DBB-Ru showed significant SOSG signal while Hf-DBA showed no singlet oxygen generation. APF and SOSG assays thus show that Hf-DBB-Ru generates both hydroxyl radical by Hf_6_ SBUs via radiosensitization and singlet oxygen through energy transfer from Hf_6_ SBUs to DBB-Ru ligands upon X-ray irradiation.

### Mitochondria targeting

We hypothesized that small size and cationic nature of Hf-DBB-Ru could lead to efficient uptake by tumor cells and subsequent internalization into mitochondria. We compared mitochondria-targeting capability of Hf-DBB-Ru to the neutral nMOF Hf-DBA. We first used inductively coupled plasma-mass spectrometry (ICP-MS) to determine time-dependent cellular uptake of Hf-DBA and Hf-DBB-Ru on MC38 cells over 8 h (Supplementary Methods and Supplementary Fig. 5). Hf-DBB-Ru was rapidly uptaken by MC38 cells, reaching saturation after 2 h incubation. At 1 h incubation, 71.3% Hf-DBB-Ru was already uptaken by MC38 cells. In comparison, only 6.8% Hf-DBA was uptaken by MC38 cells after 1 h incubation. These results indicate that MC38 cells uptake cationic Hf-DBB-Ru more efficiently than Hf-DBA with a negative ζ potential.

**Fig. 2:**
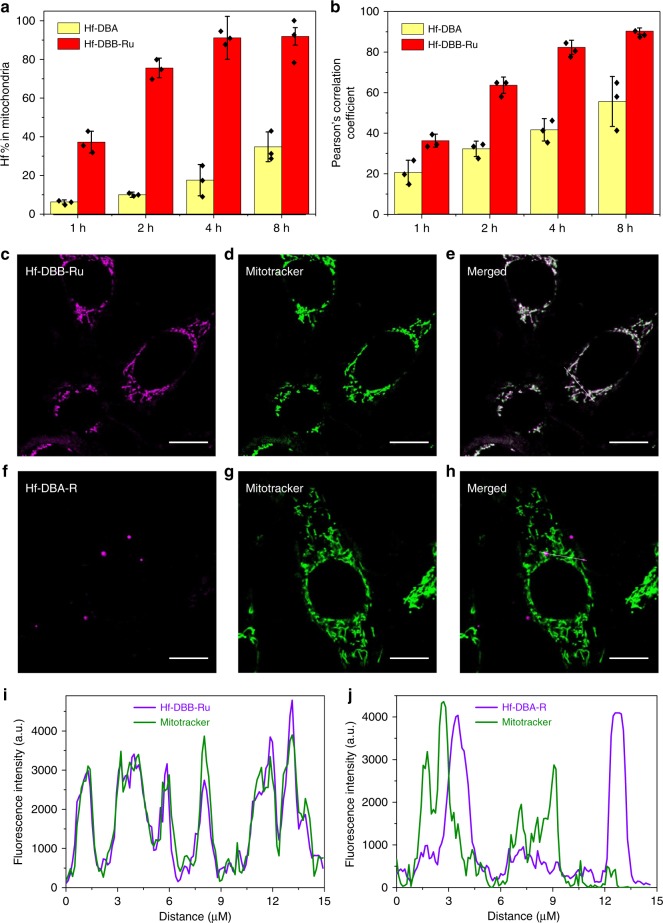
In vitro subcellular localization of Hf-DBA and Hf-DBB-Ru. **a** Time-dependent enrichment of Hf-DBA or Hf-DBB-Ru in mitochondria. Mitochondria were isolated from nMOF treated cells and the nMOF amounts were quantified by ICP-MS, *n* = 3. **b** Time-dependent Pearson’s correlation coefficients. Co-localization of Hf-DBA-R or Hf-DBB-Ru with mitochondria was analyzed based on fluorescence images captured at 1, 2, 4, and 8 h incubation. *N* = 3. Representative mitochondria co-localization images of **c**–**e** Hf-DBA-R and **f**–**h** Hf-DBB-Ru by CLSM. Scale bar = 5 µm. Mitochondria were labeled with Rhodamine 123 in green (**d**, **g**). Two nMOFs emitted magenta signal (**c**, **f**). White areas merged from the magenta and green signals represent co-localization of Hf-DBA-R or Hf-DBB-Ru with mitochondria. Fluorescence topographic profiles (**i**, **j**) display fluorescence intensity curves of straight white lines marked in **e** and **h**, respectively. The dots and error bars represent individual data points and s.d. values, respectively. The CLSM images were obtained with two repetitions to afford similar results

Mitochondria enrichment of Hf-DBA and Hf-DBB-Ru in MC38 cells was then quantitatively determined by ICP-MS and directly visualized by confocal laser scanning microscopic (CLSM) imaging. We extracted mitochondria using a modified protocol to quantify the percentage of internalized particles in mitochondria^38^. ICP-MS analyses showed that Hf-DBB-Ru was quickly enriched in mitochondria, reaching saturation after 4 h incubation (Fig. 2a). At 4 h, over 90% of Hf-DBB-Ru internalized by MC38 cells was found in mitochondria, while only 18% of internalized Hf-DBA was detected in mitochondria. Incorporation of cationic Ru(bpy)_3_^2+^ into nMOFs thus not only increases the efficiency of cellular uptake, but also endows mitochondria-targeting property.

Localization of Hf-DBB-Ru in mitochondria was further visualized by CLSM using its strong red phosphorescence at 628 nm when excited at 450 nm (Supplementary Methods). As a control, Hf-DBA-R, a fluorescent version of Hf-DBA, was synthesized by conjugating Rhodamine B to Hf-DBA via a thiourea linkage between the isothiocyanate group on Rhodamine B and the amine group of Hf-DBA^39^. ICP-MS studies indicated that Hf-DBA-R exhibited similar cellular uptake as Hf-DBA (Supplementary Fig. 6). After incubating MC38 cells with Hf-DBA-R or Hf-DBB-Ru for 1, 2, 4, and 8 h, CLSM images were captured for co-localization analysis (Supplementary Fig. 7). Hf-DBA-R and Hf-DBB-Ru showed red color while mitochondria were labeled with green Rhodamine 123 dye. The co-localization of nMOFs with mitochondria was observed as merged yellow areas. Hf-DBB-Ru-treated cells showed significantly more yellow areas than Hf-DBA-R at all timepoints and gave higher Pearson’s correlation coefficients than Hf-DBA-R after detailed analyses of CLSM images using the co-localization analysis plugin for ImageJ (Fig. 2b). To confirm the fast cellular internalization and mitochondrial enrichment of Hf-DBB-Ru, time-dependent confocal imaging studies were performed to monitor endocytosis/endosomal escape and mitochondria uptake processes. As shown in Supplementary Figs. 8, 9, internalized Hf-DBB-Ru escaped from endo/lysosome and was simultaneously enriched in mitochondria within 1 h.

To visualize co-localization between nMOFs and mitochondria at higher spatial resolutions, we performed super-resolution confocal imaging and analyzed intensity profiles by ImageJ. White signals come from merging of magenta signals from nMOFs and green signals from Rhodamine 123-labeled mitochondria. As shown in Fig. 2c–h, Hf-DBB-Ru clearly exhibited stronger white signals than Hf-DBA-R due to higher accumulation of Hf-DBB-Ru in mitochondria. The corresponding luminescence intensity profiles for Hf-DBB-Ru and Hf-DBA-R are shown in Fig. 2i, j, respectively. The magenta luminescence signal from Hf-DBB-Ru traced the green fluorescence from mitochondria (Fig. 2i), but the magenta fluorescence of Hf-DBA-R was well separated from the green fluorescence of mitochondria (Fig. 2j). Taken together, Hf-DBB-Ru was efficiently uptaken by tumor cells and internalized into mitochondria due to its small particle size and highly delocalized positive charges on the particle.

### In vitro anti-cancer efficacy via radiotherapy–radiodynamic therapy

We recently discovered that, upon X-ray irradiation, Hf-porphyrin nMOFs enhance RT by generating hydroxyl radicals and enable RDT by directly transferring energy from Hf-oxo SBUs to porphyrin ligands to sensitize the formation of ^1^O_2_ in a process termed RT-RDT^35^. We tested the ability of Hf-DBB-Ru in inducing RT-RDT by performing clonogenic assays, γ-H2AX detection, 3-(4,5-dimethylthiazol-2-yl)-5-(3-carboxymethoxyphenyl)-2-(4-sulfophenyl)-2H-tetrazolium (MTS) assay, SOSG detection, and COX-2 upregulation assays. Clonogenic assays and γ-H2AX detection evaluate the RT effects whereas MTS, SOSG, and COX-2 upregulation assays examine the RDT effects in vitro.

**Fig. 3:**
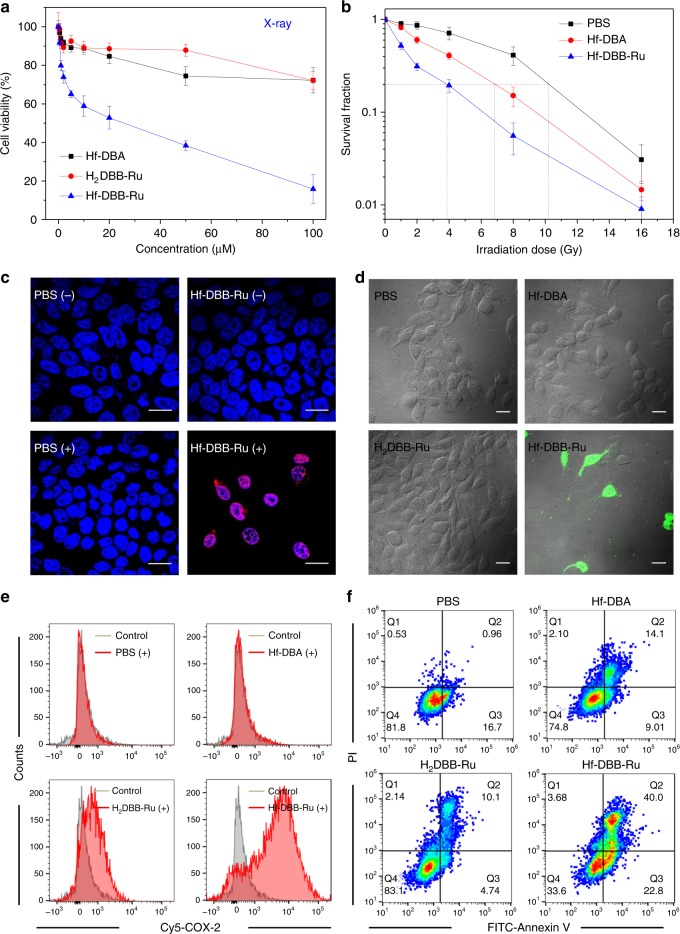
In vitro anti-cancer efficacy via RT-RDT. **a** Cytotoxicity of Hf-DBA, H_2_DBB-Ru, and Hf-DBB-Ru upon X-ray irradiation at a dose of 2 Gy in MC38 cells, *n* = 6. **b** Clonogenic assay for evaluating radioenhancement upon X-ray irradiation on MC38 cells, *n* = 6. **c** γ-H2AX assays showing DSBs in MC38 cells treated with Hf-DBB-Ru or PBS and X-ray irradiation, confirming the radioenhancing effect. Scale bar = 20 µm. (+) and (−) refer to with and without irradiation, respectively. **d** ^1^O_2_ generation was detected using SOSG in live cells by CLSM. Scale bar = 10 µm. **e** Quantification of COX-2 by flow cytometry. The cells were incubated with PBS, Hf-DBA, H_2_DBB-Ru, and Hf-DBB-Ru and irradiated with X-rays at a dose of 2 Gy. **f** Annexin V/PI analysis of MC38 cells. Cells were incubated with PBS, Hf-DBA, H_2_DBB-Ru, and Hf-DBB-Ru with and without X-ray irradiation at a dose of 2 Gy. The quadrants from lower left to upper left (counter clockwise) represent healthy, early apoptotic, late apoptotic, and necrotic cells, respectively. The percentage of cells in each quadrant is shown on the graphs. The error bars represent s.d. values. The flow cytometry studies were obtained with two repetitions to afford similar results

In vitro anti-cancer efficacy of Hf-DBA, H_2_DBB-Ru, and Hf-DBB-Ru against MC38 was first investigated by MTS assay (Fig. 3a). The H_2_DBB-Ru ligand or each of the Hf-based nMOFs was incubated with cells at various concentrations for 4 h, followed by irradiation with an X-ray irradiator at a dose of 2 Gy. Hf-DBB-Ru significantly outperformed Hf-DBA and H_2_DBB-Ru ligand in MTS assays. The IC_50_ values for Hf-DBB-Ru, Hf-DBA, and H_2_DBB-Ru against MC38 cells were calculated to be 20.13 ± 8.58, (14.91 ± 2.2) × 10^3^, and (3.69 ± 0.31) × 10^6^ μM, respectively. No cytotoxicity was observed in dark control groups (Supplementary Fig. 10).

Clonogenic assays were performed to assess the colony-forming potential of cells treated with nMOFs at a Hf concentration of 20 µM for 4 h followed by irradiation with X-ray from 0 to 16 Gy. Treated cells were trypsinized, re-seeded, and cultured for 15 days and the clones were counted and plotted with the survival fraction as shown in Fig. 3b. A radiation enhancement factor at 10% survival dose (REF_10_) was calculated as the ratio of equivalent irradiation doses needed to give 10% survival rate for the PBS control group over that for the experimental groups. At the same Hf concentration, Hf-DBB-Ru outperformed Hf-DBA in radiosensitization, with an REF_10_ value of 2.68 compared to 1.50 for Hf-DBA. Interestingly, different from clonogenic curves of conventional radiosensitizers, the concave-shaped dose-response curves of Hf-DBB-Ru confirmed early response of cells to ^1^O_2_ due to the RDT effect^35^.

To confirm the radiosensitization effect of Hf-DBB-Ru, we determined DNA double-strand breaks (DSBs) by detecting phosphorylated γ-H2AX, a sensitive protein biomarker for DSB repair induced by ionizing radiation. When incubated with Hf-DBB-Ru and irradiated with X-rays at a dose of 2 Gy, significant γ-H2AX fluorescence indicating DSBs was observed in the nuclei of MC38 cells (Fig. 3c), similar to the red fluorescence observed for Hf-DBA treated cells upon X-ray irradiation (Supplementary Fig. 11). No DSB was observed in PBS or H_2_DBB-Ru-treated cells with irradiation or any particle treated cells without X-ray irradiation. These results indicate that Hf-DBB-Ru exhibits strong radiosensitization effect to cause DNA DSBs.

MC38 cells treated with Hf-DBB-Ru and X-ray irradiation followed by incubation with SOSG showed strong green fluorescence by CLSM, indicating the generation of ^1^O_2_ (Fig. 3d & Supplementary Fig. 12). No fluorescence was detected in PBS, Hf-DBA, and H_2_DBB-Ru-treated group with or without X-ray irradiation or in Hf-DBB-Ru treatment without irradiation. ^1^O_2_ was only generated intracellularly by combining Hf-DBB-Ru and X-ray irradiation, suggesting that heavy metal SBUs, photosensitizers, and X-rays are three indispensable elements for the generation of ^1^O_2_ in nMOF-enabled RDT. It is worth noting that the H_2_DBB-Ru-treated group showed weak green signals, likely due to direct excitation of H_2_DBB-Ru from slight absorption of X-rays by Ru atoms.

COX-2, a cyclooxygenase involved in lipid peroxidation, is up-regulated after ^1^O_2_-induced cell membrane damage, which is commonly observed after PDT treatment^40^. We determined the upregulation of COX-2 in MC38 cells after PBS, Hf-DBA, H_2_DBB-Ru, or Hf-DBB-Ru incubation with and without X-ray irradiation by quantitative flow cytometric analysis. 24 h after irradiation, treated cells were collected, fixed and stained with biotin-conjugated COX-2 antibody and followed by incubation with Cy5-conjugated streptavidin. The intensity shift of red fluorescence compared to the control indicated COX-2 upregulation of cells. No COX-2 upregulation was observed from PBS or Hf-DBA treated group or dark controls (Supplementary Fig. 13). As shown in Fig. 3e, the mean fluorescence intensities were 8973, 1356, 231, and 186 for the cells treated with Hf-DBB-Ru, H_2_DBB-Ru, Hf-DBA and PBS with X-ray irradiation, respectively. This result is consistent with SOSG assay showing strong RDT effects by Hf-DBB-Ru and low level ^1^O_2_ generation by H_2_DBB-Ru from slight absorption of X-rays by Ru atoms.

The cell death pathways were then evaluated with an Annexin V/Cell death kit. As shown in Fig. 3f, significant amounts of cells underwent apoptosis/necrosis when treated with Hf-DBB-Ru and X-ray irradiation, with only 7.23% healthy cells, compared to 75.7% and 82.9% healthy cells for Hf-DBA or H_2_DBB-Ru plus X-ray irradiation, respectively. > 90% cells remained healthy in dark controls and in the PBS group with irradiation, indicating that Hf-DBA, H_2_DBB-Ru, and Hf-DBB-Ru are not intrinsically cytotoxic and the low dose X-ray elicited negligible cytotoxicity with PBS treatment (Supplementary Figs. 14, 15). Furthermore, severe morphological changes were observed for cells treated with Hf-DBB-Ru plus X-ray irradiation (Supplementary Methods and Supplementary Fig.16). Taken together, Hf-DBB-Ru possesses a strong cell killing effect when combined with X-ray irradiation due to the RT-RDT effects.

### In vitro mechanistic studies of Mitochondria-targeted RT-RDT

In PDT, subcellular localization of a PS determines the initial photodynamic damage and ensuing cellular responses. The results from the proceeding sections suggested the enhanced RT-RDT efficacy of Hf-DBB-Ru due to its mitochondria-targeting property. The generation of ^**·**^OH and ^1^O_2_ in mitochondria can cause maximal membrane damage to initiate an efficient apoptotic process. To gain mechanistic insights into mitochondria-targeted RT-RDT, we evaluated depolarization of the mitochondria membrane potential, the release of cytochrome c, and the loss of respiratory chain activity in MC38 cells following Hf-DBB-Ru treatment and X-ray irradiation.

**Fig. 4:**
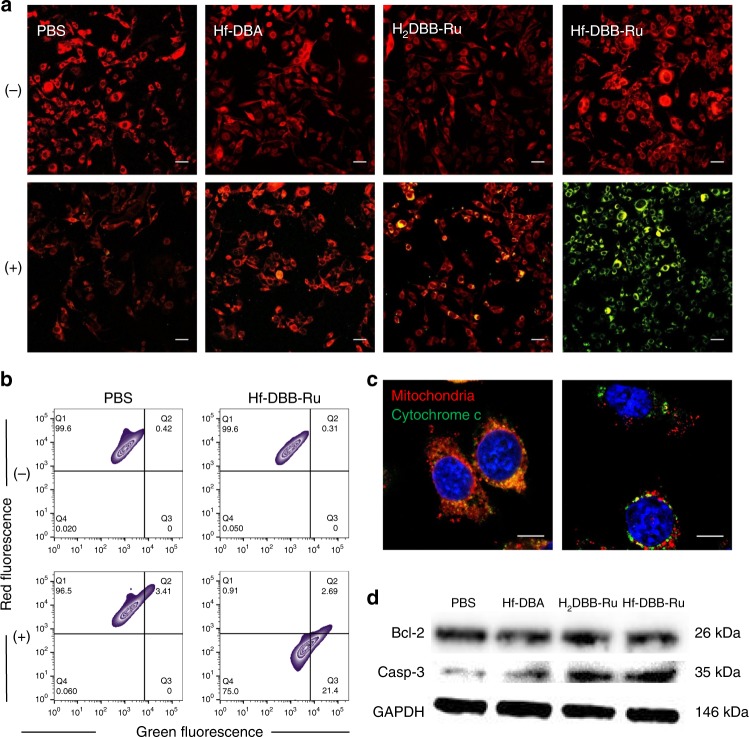
In vitro mechanistic studies of mitochondria-targeted RDT. **a** Fluorescence images of MC38 cells stained with JC-1 4 h after treatment of PBS, Hf-DBA, H_2_DBB-Ru and Hf-DBB-Ru with (2 Gy, + ) or without (-) X-ray irradiation. Green fluorescence indicates the depolarization of mitochondrial membrane potential. Scale bar = 50 µm. **b** Flow cytometric analysis of green versus red fluorescence of JC-1-stained MC38 cells 4 h after treatment of PBS and Hf-DBB-Ru with (2 Gy, + ) or without (-) X-ray irradiation. **c** cytochrome c released from mitochondria 8 h after treatment of PBS (left) or Hf-DBB-Ru (right) upon X-ray irradiation (2 Gy). Green: FITC-conjugated cytochrome c antibody; Red: Mitotracker CMXRos; Blue: DAPI. Scale bar = 10 µm. **d** Bcl-2 and Caspase-3 protein expression levels of MC38 cells 8 h after treatment of PBS, Hf-DBA, H_2_DBB-Ru and Hf-DBB-Ru upon X-ray irradiation (2 Gy). The glyceraldehyde 3-phosphate dehydrogenase (GAPDH) band served as loading control. The flow cytometry and CLSM studies were obtained with two repetitions to afford similar results

The depolarization of mitochondrial membrane potential was assayed with the mitochondria permeable dye 5,5’,6,6’-tetrachloro-1,1’,3,3’-tetraethylbenzimidazolylcarbocyanine iodide (JC-1). With a polarized mitochondrial potential in normal cells, JC-1 emits red fluorescence from the J-aggregate that forms in the mitochondrial matrix. When the mitochondrial membrane potential depolarizes, JC-1 exists as a monomer and emits green fluorescence. The shift from red to green fluorescence is thus an indicator for a decrease in the mitochondrial membrane potential. MC38 cells treated with Hf-DBA, H_2_DBB-Ru, or Hf-DBB-Ru at an equivalent dose of 20 µM of Hf upon X-ray irradiation at a dose of 0 or 2 Gy were stained with JC-1 for CLSM analyses. Cells treated with PBS without X-ray irradiation served as control. As shown in Fig. 4a and Supplementary Fig. 17, all four groups showed strong red fluorescence and negligible green fluorescence without X-ray treatment. 4 h after X-ray irradiation, obvious green fluorescence was observed in Hf-DBB-Ru-treated group, while Hf-DBA and H_2_DBB-Ru-treated groups only showed a few yellow spots, indicating only weak green fluorescence. Flow cytometry analyses further confirmed these observations. Upon X-ray irradiation, the Hf-DBB-Ru group showed an increased number of mitochondria with green fluorescence and few mitochondria with red fluorescence. In contrast, the groups treated with PBS, Hf-DBA, or H_2_DBB-Ru did not show such a distinct change upon X-ray irradiation (Fig. 4b and Supplementary Fig. 18). The depolarization of mitochondria membrane potential is an indicator of mitochondria dysfunction^41^. With an increase in ROS generation and a decrease in ATP synthesis, the permeability transition pore complex is primed to release cytochrome c into the cytosol to form a complex with cytoplasmic apoptosis activating factor-1, which activates caspase-9 and caspase-3 to induce apoptosis. Mitochondria and cytochrome c were stained with Mitotracker and FITC-conjugated cytochrome c antibody, respectively, 6 h after X-ray irradiation for CLSM imaging. Compared with other groups, the Hf-DBB-Ru-treated group displayed an obvious separation of green and red fluorescence (Fig. 4c and Supplementary Fig. 19), indicating the release of cytochrome c into cytosol.

To probe if the anti-cancer effect originates from RT-RDT damage to mitochondria, we detected Bcl-2 and caspase-3 protein expression levels 8 h after X-ray irradiation. PBS, Hf-DBA, H_2_DBB-Ru, and Hf-DBB-Ru-treated cells all showed similar levels of Bcl-2 protein based on western blots (Fig. 4d), indicating no strong downregulation of Bcl-2 protein expression among all groups, likely due to local RT-RDT effects causing mitochondria outer membrane permeabilization. In contrast, Hf-DBB-Ru-treated cells showed upregulation of caspase-3 in comparison to PBS, Hf-DBA, or H_2_DBB-Ru-treated groups (Fig. 4d). Bcl-2 and caspase-3 quantification results thus indicated that the RT-RDT damage was initiated from mitochondria to directly induce the downstream apoptotic pathways without downregulating Bcl-2 due to the localization of Hf-DBB-Ru in mitochondria^42^.

The dysfunction of mitochondria led to the loss of respiratory chain activity and increased generation of reduced nicotinamide adenine dinucleotide phosphate (NADPH). We evaluated the respiratory chain activity and viability by (3-(4,5-Dimethylthiazol-2-yl)-2,5-diphenyltetrazolium bromide (MTT) assay (Supplementary Methods). In this assay, MTT is reduced to formazan, which appears as dark blue microgranules^43^ in cells with normal respiratory chain activity (Supplementary Fig. 20). The amount of formazan correlates with the activity of the respiratory chain. Compared with control groups, the Hf-DBB-Ru plus X-ray group showed significantly decreased amount of reduced formazan, suggesting the loss of the respiratory chain activity after mitochondria-targeted RT-RDT treatment.

### In vivo anti-tumor efficacy of mitochondria-targeted RT-RDT

**Fig. 5:**
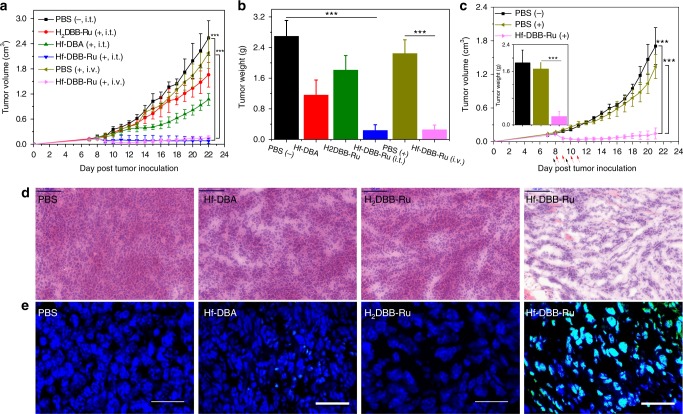
In vivo anti-cancer efficacy of mitochondria-targeted RDT. **a** Tumor growth inhibition/regression curves in MC38 tumor-bearing mice treated with PBS, Hf-DBA, DBB-Ru, or Hf-DBB-Ru by intratumoral (i.t.) injection with (+) or without (−) X-ray irradiation or Hf-DBB-Ru by intravenous (i.v.) injection followed by X-ray irradiation. *n* = 6. **b** Excised tumor weights on day 22. *n* = 6. **c** Tumor growth inhibition/regression curves in CT26 tumor-bearing mice treated with PBS with (+) or without (−) X-ray irradiation or i.v. injected with Hf-DBB-Ru followed by X-ray irradiation. Black arrows refer to i.v. injection of different treatments and red arrows refer to X-ray irradiation. Excised tumor weights on day 21 shown in the inset. *n* = 6. **P < 0.05, ***P < 0.001 from control by *t*-test. Representative (**d**) H&E histological staining and (**e**) TUNEL immunofluorescence staining of excised tumor slices for PBS, Hf-DBA, H_2_DBB-Ru, and Hf-DBB-Ru (i.t. injection) treatment groups (left to right), respectively. **d** H&E, scale bar = 100 μm. **e** TUNEL, scale bar = 50 μm. The error bars represent s.d. values. The in vivo confocal images were obtained without repetition

A colorectal adenocarcinoma mouse model of MC38 tumor-bearing C57BL/6 mice was employed to evaluate the anti-tumor efficacy of Hf-DBA and Hf-DBB-Ru in vivo. The H_2_DBB-Ru ligand and PBS served as controls. When the tumors reached 100–150 mm^3^ in volume on day 7 post tumor inoculation, Hf-DBB-Ru, Hf-DBA, or H_2_DBB-Ru was injected intratumorally at equivalent doses of 0.2 µmol per mouse followed by daily X-ray irradiation at a dose of 1 Gy per fraction (225 kVp, 13 mA, 0.3 mm Cu filter) for a total of 6 fractions on consecutive days. Tumor sizes and body weights were measured daily. All mice were sacrificed 22 days after tumor inoculation, and the excised tumors were photographed and weighed. As shown in Fig. 5a, the H_2_DBB-Ru-treated group showed slight tumor growth inhibition with a T/C ratio of 65.6% on Day 22, which was likely due to the RT-RDT damage to mitochondria caused by slight X-ray absorption by the Ru compound. The Hf-DBA group showed moderate tumor suppression with a T/C ratio of 42.1%, indicating the radiosensitization effect of Hf-DBA. In stark contrast, Hf-DBB-Ru treatment led to effective tumor regression at a low X-ray dose of 6 Gy. The average tumor volume of the Hf-DBB-Ru treatment group was only 3.0 % of that of the PBS control on Day 22, affording an impressive T/C ratio of 3.0 %.

We also tested the therapeutic efficacy of intravenously injected Hf-DBB-Ru followed by X-ray irradiation. Hf-DBB-Ru was injected intravenously to MC38 tumor-bearing mice at a dose of 2 µmol per mouse twice on day 7 and day 9 followed by daily X-ray irradiation at a dose of 2 Gy per fraction for a total of four consecutive fractions on days 8–11. PBS injected intravenously followed the same X-ray irradiation treatment served as a control. Tumor sizes and body weights were measured daily. As shown in Fig. 5a, the PBS group treated with daily X-ray irradiation did not exhibit statistically significant difference from the untreated group, indicating that fractionated X-ray irradiation for a total dose of 8 Gy does not have radiotherapeutic effects. In contrast, intravenously injected Hf-DBB-Ru followed by X-ray irradiation led to effective tumor regression with a T/C ratio of 6.1%. There is no statistically significant difference between the tumors of the mice intratumorally injected with Hf-DBB-Ru followed by X-ray irradiation and those of the mice intravenously injected with Hf-DBB-Ru followed by X-ray irradiation.

We confirmed the radiotherapeutic efficacy of intravenously injected Hf-DBB-Ru followed by X-ray irradiation on BALB/c mice bearing CT26 tumors, another commonly used colorectal adenocarcinoma mouse model. Similar tumor regression results were observed on the CT26 with a T/C ratio of 8.5% (Fig. 5c) when the mice were intravenously injected with Hf-DBB-Ru at a dose of 2 µmol per mouse twice on day 7 and day 9 followed by daily X-ray irradiation at a dose of 2 Gy per fraction for a total of four consecutive fractions on days 8–11. These results indicate that Hf-DBB-Ru-enabled mitochondria-targeted RT-RDT can be administered to treat many different types of cancers. Statistical analysis of tumor sizes of all groups is listed in Supplementary Table 3.

The tumor growth inhibition/regression results of MC38 model were confirmed by the weights of excised tumors on Day 22 (Fig. 5b and Supplementary Figs. 21–23). The weights of tumors treated with i.t. and i.v. injected Hf-DBB-Ru were 0.23 ± 0.16 and 0.25 ± 0.13 g compared to 2.69 ± 0.42 g, 1.15 ± 0.40 g, and 1.80 ± 0.39 g for tumors treated with PBS, Hf-DBA, and H_2_DBB-Ru, respectively. For CT26 model, the weights of excised tumors on Day 21 treated with i.v. injected Hf-DBB-Ru was 0.25 ± 0.17 g compared to 1.67 ± 0.30 g and 1.85 ± 0.38 g for tumors treated with PBS with or without X-ray irradiation, respectively. Statistical analysis of tumor weights of all groups is listed in Supplementary Table 4. H&E histological staining indicated severe necrosis of tumor slices from Hf-DBB-Ru treatment (Fig. 5d). A TdT-mediated dUTP nick end labeling (TUNEL, Invitrogen, USA) assay was performed to further evaluate the in vivo apoptosis. The sectioned tumor slices were fixed with acetone and then stained sequentially according to the product protocol. DAPI was employed to label cell nuclei. As shown in Fig. 5e, strong green fluorescence was observed in Hf-DBB-Ru-treated tumor slice, indicating significant apoptosis after treatment. In comparison, negligible green fluorescence was found in H_2_DBB-Ru, Hf-DBA and PBS treated groups. The staining results support the high anti-tumor efficacy from mitochondria-targeted RT-RDT by Hf-DBB-Ru. Finally, steady body weights, similar weight gain patterns, and no obvious difference in behaviors and organ functions were observed in all groups, indicating that intratumoral administration of mitochondria-targeted Hf-DBB-Ru was not systemically toxic (Supplementary Figs. 24–26). The lack of abnormalities on histological images of frozen major organ slices further supported non-toxic nature of Hf-DBB-Ru-enabled RT-RDT treatment (Supplementary Fig. 27).

## Discussion

**Fig. 6:**
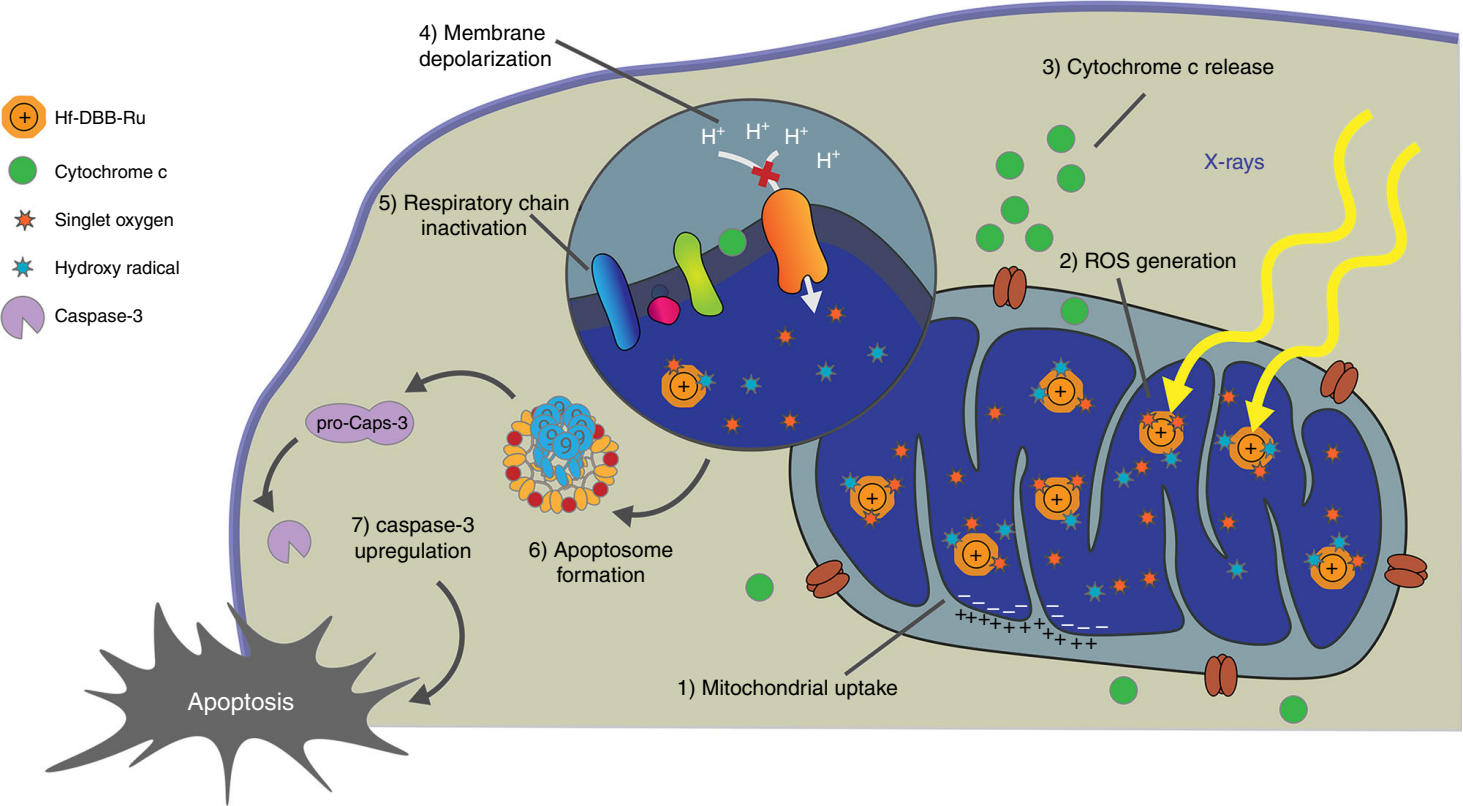
Mitochondria-targeted RT-RDT mediated by Hf-DBB-Ru. Hf-DBB-Ru was internalized by tumor cells efficiently and enriched in mitochondria due to dispersed cationic charges in the nMOF framework. Hf_6_ SBUs preferentially absorb X-rays over tissues to enhance RT by sensitizing hydroxyl radical generation and enable RDT by transferring energy to Ru(bpy)_3_^2+^-based bridging ligands to generate singlet oxygen. The RT-RDT process trigger mitochondrial membrane potential depolarization, membrane integrity loss, respiratory chain inactivation, and cytochrome c release to initiate apoptosis of cancer cells

In this work we report a new strategy to realize mitochondria-targeted therapy using low doses of highly penetrating X-rays (Fig. 6). Considering mitochondria-targeting property of positively charged Ru(bpy)_3_^2+^, we synthesized a UiO-like nMOF, Hf-DBB-Ru, by incorporating Ru(bpy)_3_^2+^ moieties into the bridging ligand. We demonstrated mitochondria-targeting property of Hf-DBB-Ru by ICP-MS quantification, time-dependent confocal imaging, and super-resolution imaging. The time-dependent process of mitochondria damage-initiated apoptotic cell death was mapped with ^1^O_2_ generation, mitochondria membrane depolarization, cytochrome c release, respiratory chain disruption, caspase-3 expression, COX-2 upregulation, and annexin V assays.

Two major classes of compounds have been explored for mitochondria targeting^44^. The first class of compounds use specific functionalities to target mitochondrial lipids, proteins, or receptors, such as cardiolipin and benzodiazepine receptors^12,45,46^. For instance, 10-N-nonyl acridine orange is known as a specific marker for cardiolipin and used for probing mitochondrial membrane independent of their energized state^47^. The second class of compounds contain lipophilic cationic moieties, called delocalized lipophilic cations (DLCs), such as triphenylphosphonium^20,48^, which selectively accumulate in mitochondria with the help of mitochondrial membrane potential. Mitochondria have a distinctly negative membrane potential of up to −180 mV, leading to preferential accumulation of cationic species in mitochondria^49^. According to the Nernst Equation, every −60 mV membrane potential gives a tenfold accumulation of positively charged monovalent molecules^50^. Highly lipophilic photosensitizers with delocalized cationic charges can accumulate 1000 times more in the mitochondria compared to the cytoplasm. A number of DLC containing dyes, such as Rhodamine 123 and JC-1, have been developed for mitochondrial staining^50^. Triphenylphosphonium has been utilized as a mitochondria-targeting moiety in various cancer treatments to improve anti-tumor efficacy^48,51,52^.

DLCs are positively charged molecules that possess delocalized electronic structures through resonance stabilization. The transport of these charged lipophilic molecules though mitochondrial membrane is energetically favorable due to two factors. First, the charge is spread over a large molecular area, which effectively lowers the enthalpy associated with desolvating charged species and placing them into a lipid environment^44^. Second, the relatively dispersed positive charge reduces repulsive interaction with cationic species inside the mitochondrial intermembrane space when entering the mitochondrial bilayer membrane. As a result, DLCs are driven by membrane potentials to preferentially localize in the mitochondrial matrix. We showed that the incorporation of cationic Ru(bpy)_3_^2+^ species endows the nMOF with a large, dispersed positive charge for highly effective mitochondria targeting. Toxicity is a potential concern for DLCs as they have been shown to compromise mitochondrial function at high concentrations^53^. However, we did not observe any cytotoxicity in Hf-DBB-Ru-treated cells without X-ray irradiation. With a high stability in physiological environments, Hf-DBB-Ru represents a novel class of mitochondria-targeting agents without dark toxicity.

Ru(bpy)_3_^2+^ is an excellent photosensitizer for singlet oxygen generation, outperforming porphyrin and its derivatives in lifetimes and ^1^O_2_ quantum yields. However, due to large Stokes shifts, Ru(bpy)_3_^2+^ and related metal-containing PSs must be excited with short-wavelength photons which have a poor tissue penetration. By incorporating Ru(bpy)_3_^2+^ into a Hf-based nMOF, we have developed a novel radioenhancer via the RT-RDT mechanism. Upon irradiation with low-dose X-rays, the Hf_6_ SBUs in Hf-DBB-Ru efficiently absorb X-rays to not only enhance RT via hydroxyl radical generation but also enable RDT via exciting Ru(bpy)_3_^2+^ to generate ^1^O_2_. In vitro MTS and colony assays demonstrated superior cytotoxicity of Hf-DBB-Ru mediated RT-RDT. The in vivo anti-cancer efficacy of this nMOF-enabled, mitochondria-targeted RT-RDT was further demonstrated on two mouse models of colorectal tumors.

In summary, we have designed the first nMOF for mitochondria-targeted and X-ray activated cancer therapy. The integration of Ru(bpy)_3_^2+^ photosensitizers into Hf-DBB-Ru led to a nMOF with strong mitochondria-targeting property. When irradiated with low doses of highly penetrating X-rays, Hf-DBB-Ru-enabled RT-RDT by efficiently generating hydroxyl radicals from the Hf_6_ SBUs and singlet oxygen from the DBB-Ru PSs. The mitochondria-targeted RT-RDT process depolarized the mitochondrial membrane potential, released cytochrome c, and disturbed the respiratory chain to initiate apoptotic pathways for programmable cell death. The nMOF-enabled, mitochondria-targeted RT-RDT significantly regressed colorectal tumors on mouse models at very low X-ray doses and with no side effects.

## Methods

### Cell line and animals

The murine colon adenocarcinoma cell lines, MC38 and CT26, was purchased from the American Type Culture Collection (Rockville, MD, USA). MC38 cells were cultured in Dulbecco’s Modified Eagle’s Medium (DMEM) medium (GE Healthcare, USA) supplemented with 10% fetal bovine serum (FBS, VWR, USA). CT26 cells were cultured in Roswell Park Memorial Institute (RPMI) 1640 medium (GE Healthcare, USA) supplemented with 10% FBS. Medium was further supplemented with 100 U per mL penicillin G sodium and 100 μg per mL streptomycin sulfate. Cells were cultured in a humidified atmosphere containing 5% CO_2_ at 37 °C. C57Bl/6 and Balb/c mice (6–8 weeks) were obtained from Harlan-Envigo Laboratories, Inc (USA). Mycoplasma was tested before use by MycoAlert detection kit (Lonza Nottingham, Ltd.) The study protocol was reviewed and approved by the Institutional Animal Care and Use Committee (IACUC) at the University of Chicago.

### Synthesis of Hf-DBB-Ru nMOF

To a 1 dram glass vial was added 0.5 mL of HfCl_4_ solution (2.0 mg per mL in DMF), 0.5 mL of H_2_DBB-Ru solution (4.0 mg per mL in DMF), 5 μL of trifluoroacetic acid and 2 μL of water. The reaction mixture was kept in an 80 °C oven for 24 h. The orange precipitate was collected by centrifugation and washed with DMF and ethanol. Yield: 78.4%.

### Synthesis of Hf-DBA nMOF

Hf-DBA nMOFs were synthesized according to the reported protocol^54^. To a 1 dram glass vial was added 0.5 mL of HfCl_4_ solution (2.0 mg per mL in DMF), 0.5 mL of the 2,5-di(p-benzoato)aniline (H_2_DBA) solution (2.0 mg per mL in DMF) and 0.5 μL of trifluoroacetic acid. The reaction mixture was kept in a 60 °C oven for 72 h. The white precipitate was collected by centrifugation and washed with DMF and ethanol. Yield: 44.1%.

### Hydroxyl radical detection

Hf-DBA and Hf-DBB-Ru were suspended in water at equivalent Hf concentrations of 20 μM in the presence of 5 μM APF. A water solution of 5 μM APF was used as a control for background subtraction. 100 μL of each suspension was added to a 96-well plate and then irradiated with 0, 1, 2, 3, 5, or 10 Gy X-ray (Philips RT250 X-ray generator, Philips, USA, 250 KVp, 15 mA, 1 mm Cu filter). The fluorescence signal was immediately collected with the Xenogen IVIS 200 imaging system.

### Singlet oxygen detection

Hf-DBA and Hf-DBB-Ru were suspended in water at equivalent Hf concentrations of 20 μM in the presence of 12.5 μM SOSG. A water solution of 12.5 μM SOSG was used as a control for background subtraction. 100 μL of each suspension was added to a 96-well plate and then irradiated with 0, 1, 2, 3, 5, or 10 Gy X-ray (Philips RT250 X-ray generator, Philips, USA, 250 KVp, 15 mA, 1 mm Cu filter). The fluorescence signal was immediately collected with the Xenogen IVIS 200 imaging system.

### Synthesis of Hf-DBA-R

The Hf-DBA nMOF was dispersed in DMF (1 mL, 1 mmol per L by DBA concentration). To the dispersion was added 25 μL of rhodamine B isothiocyanate solution (2 mmol per L in DMF). The mixture was stirred overnight (in the dark). The resulting Hf-DBA-R nMOF was washed with ethanol and water.

### Co-localization of nMOFs and Mitochondria

MC38 cells were cultured in 35 mm tissue culture dishes overnight and incubated with Hf-DBA-R or Hf-DBB-Ru at an equivalent dose of 20 µM for 4 h. Cellular nuclei and mitochondria were labeled with Hoechest 33342 and Rhodamine 123, respectively. The slides were then washed with PBS and observed under CLSM. Fluorescence topographic profiles and Pearson’s correlation coefficients were obtained using the Co-localization Analysis plugin for ImageJ^55^.

### Isolation of mitochondria

Extraction of mitochondria was conducted according to previously reported protocol, with a few modifications^38^. MC38 cells were washed twice in mitochondrial extraction buffer containing mannitol (200 mM), sucrose (70 mM), HEPES (10 mM), and EGTA (1.0 mM) at pH 7.2 and 4 ℃ and then resuspended for homogenization. The homogenate was spun for 10 min at 600 g to recover the supernatant. The supernatant was further spun for 10 min at 11,000×*g* to recover the mitochondrial fraction for ICP-MS quantification.

### Cytotoxicity

The cytotoxicity of Hf-DBA, H_2_DBB-Ru or Hf-DBB-Ru was evaluated with MTS assay (Promega, USA) upon X-ray irradiation. MC38 cells were seeded on 96-well plates at 2 × 10^4^ per well and further cultured for 12 h. Hf-DBA, DBB-Ru or Hf-DBB-Ru were added to the cells at an equivalent ligand dose of 0, 0.5, 1, 2, 5, 10, 20, 50 and 100 μM and incubated for 4 h. The cells were then irradiated with X-rays at a dose of 2 Gy (250 kVp, 15 mA, 1 mm Cu filter). The cells were further incubated for 72 h before determining the cell viability by MTS assay.

### Clonogenic assay

The clonogenic assay was performed according to a previously reported protocol, with a few modifications^54^. MC38 cells were cultured in a 6-well plate overnight and incubated with particles at a Hf concentration of 20 µM for 4 h followed by irradiation with 0, 1, 2, 4, 8 and 16 Gy X-ray (250 kVp, 15 mA, 1 mm Cu filter). Cells were trypsinized and counted immediately. 100–1000 cells were seeded in a 6-well plate and cultured with 2 mL medium for 10–20 days. Once colony formation was observed, the culture medium was discarded. The plates were rinsed twice with PBS, then stained with 500 µL of 0.5% w/v crystal violet in 50% methanol per H_2_O. The wells were rinsed with water and the colonies were counted manually.

### In vitro singlet oxygen generation

SOSG reagent (Life Technologies, USA) was employed for the detection of singlet oxygen. MC38 cells were seeded on cover slides in 35 mm tissue culture dishes overnight. Hf-DBA, H_2_DBB-Ru or Hf-DBB-Ru was added to the cells at an equivalent dose of 20 μM. Cells incubated with PBS served as a control. After incubation of 4 h, cells were irradiated with X-rays (250 kVp, 15 mA, 1 mm Cu filter) at a dose of 0 or 2 Gy. The slides were then immediately washed with PBS and observed under CLSM (FV1000, Olympus, Japan).

### COX-2 assay

The cell membrane damage caused by RDT upon X-ray was investigated by COX-2 assay (BD Bioscience, USA). MC38 cells were seeded on cover slides in 3.5 cm petri dishes and cultured for 12 h then incubated with particles at an equivalent concentration of 20 µM for 4 h followed by X-ray irradiation at 0 and 2 Gy dose. Cells were fixed with 4% paraformaldehyde 24 h after X-ray irradiation. Biotin-conjugated anti-COX-2 antibody (PerkinElmer, USA) with concentration of 10 µg per mL was incubated with cells at 4 °C overnight then followed by incubation with Cy5-conjugated streptavidin for flow cytometry. Representative gating strategy is shown in Supplementary Fig. 28 (a).

### Apoptosis/necrosis

MC38 cells were cultured in 35 mm tissue culture dishes overnight and incubated with Hf-DBA, H_2_DBB-Ru or Hf-DBB-Ru at an equivalent dose of 20 µM for 4 h followed by irradiation with 0 or 2 Gy X-ray (250 kVp, 15 mA, 1 mm Cu filter). After 24 h, the cells were stained according to the AlexaFluor 488 Annexin V/dead cell apoptosis kit (Life technology, USA) and quantified by flow cytometry with three different runs for statistical analysis. Representative gating strategy is shown in Supplementary Fig. 28 (b).

### DNA damage

MC38 cells were cultured in 35 mm tissue culture dishes overnight and incubated with particles at a Hf concentration of 20 µM for 4 h followed by irradiation at 0 and 2 Gy X-ray (250 kVp, 15 mA, 1 mm Cu filter). Cells were stained immediately with the HCS DNA damage kit (Life Technology, USA) for CLSM.

### JC-1 staining

MC38 cells were cultured in 35 mm tissue culture dishes overnight and then treated with PBS, Hf-DBA, H_2_DBB-Ru, or Hf-DBB-Ru at an equivalent dose of 20 µM for 4 h followed by irradiation with 0 or 2 Gy X-ray (250 kVp, 15 mA, 1 mm Cu filter). 4 h later, samples were stained with JC-1 (Abcam, UK) at a concentration of 10 µM at 37 °C for 30 min in the dark. For CLSM, slides were washed with PBS and observed directly. For flow cytometry, cells were trypsinized and washed with PBS for analysis. Representative gating strategy is shown in Supplementary Fig. 28 (c).

### Cytochorome c release

MC38 cells were cultured in 35 mm tissue culture dishes overnight and then treated with PBS, Hf-DBA, H_2_DBB-Ru, or Hf-DBB-Ru at an equivalent dose of 20 µM for 4 h followed by irradiation with 0 or 2 Gy X-ray (250 kVp, 15 mA, 1 mm Cu filter). Cells were then stained with Mitotracker Red CMXRos (Thermofisher, USA) at a concentration of 1 µg per mL at 37 °C for 10 min, fixed and permeabilized 8 h after irradiation. After being stained with FITC-conjugated cytochrome c antibody (Thermofisher, USA) at a concentration of 10 µM at 37 °C for 1 h and DAPI, slides were observed under CLSM. The deco-localization of green and red fluorescence indicates the release of cytochrome c from mitochondria.

### Caspase-3 and Bcl-2 western blot

MC38 cells were cultured in 35 mm tissue culture dishes overnight and then treated with PBS, Hf-DBA, H_2_DBB-Ru, or Hf-DBB-Ru at an equivalent dose of 20 µM for 4 h followed by irradiation with 2 Gy X-ray (250 kVp, 15 mA, 1 mm Cu filter). After 8 h, cells were lysed and separated by SDS/polyacrylamide-gel electrophoresis. After membrane transfer, anti-caspase-3 (#9662, Cell Signaling, USA, 1: 2000) and anti-Bcl-2 (Bcl-2–100, Thermofisher, USA, 1: 1000) were separately incubated with the cropped membranes for blotting. Uncropped blots of Caspase-3 and Bcl-2 with markers are shown in Supplementary Fig. 29.

### In vivo anti-cancer efficacy

For the evaluation of RT-RDT efficacy of Hf-DBA, H_2_DBB-Ru, and Hf-DBB-Ru, a sygneneic model was established by subcutaneously inoculating 2 × 10^6^ MC38 cells onto the right flanks subcutaneous tissues of C57Bl/6 mice on day 0 as a MC38 model. When the tumors reached 100–150 mm^3^ in volume, Hf-DBA, H_2_DBB-Ru, or Hf-DBB-Ru at an equivalent dose of 0.2 µmol or PBS was injected intratumorally. 12 h after injection, mice were anaesthetized with 2% (v/v) isoflurane and the primary tumors were irradiated with 1 Gy X-ray per fraction (225 kVp, 13 mA, 0.3 mm-Cu filter) for a total of 6 daily fractions. The tumor sizes were measured with a caliper every day where tumor volume equals (width^2^ × length)/2. Body weight of each group was monitored every day. Mice were killed on Day 22 and the excised tumors were photographed and weighed. Tumors and major organs were sectioned for hematoxylin–eosin staining (H&E) and immunofluorescence analysis.

### TUNEL Assay

TdT-mediated dUTP nick end labeling (TUNEL, Invitrogen, USA) assay was used for evaluating the in vivo apoptosis. The sectioned tumor slices were fixed with acetone then stained sequentially according to product protocol. DAPI was employed to label cell nuclei.

### Statistical analysis

Group sizes (*N* > 5) were chosen to ensure proper statistical ANOVA analysis for efficacy studies. Student’s *t*-tests were used to determine if the variance between groups is similar. Statistical analysis was performed using OriginPro (OriginLab Corp.). Statistical significant was calculated using two-tailed Student’s *t*-tests and defined as *P < 0.05, **P < 0.01, ***P < 0.001. Animal experiments were not performed in a blinded fashion and are represented as mean ± SD. The co-localization analysis was performed in a blinded fashion and are represented as mean ± SD.

## Electronic supplementary material

Supplementary Information

## Data Availability

The authors declare that all the data supporting the findings of this study are available within the article and its Supplementary Information files or from the corresponding author upon reasonable request.

## References

[1] Castano AP, Mroz P, Hamblin MR (2006). Photodynamic therapy and anti-tumour immunity. Nat. Rev. Cancer.

[2] Celli JP (2010). Imaging and photodynamic therapy: mechanisms, monitoring, and optimization. Chem. Rev..

[3] Lovell JF (2011). Porphysome nanovesicles generated by porphyrin bilayers for use as multimodal biophotonic contrast agents. Nat. Mater..

[4] Huang P (2012). Light‐triggered theranostics based on photosensitizer‐conjugated carbon dots for simultaneous enhanced‐fluorescence imaging and photodynamic therapy. Adv. Mater..

[5] Agostinis P (2011). Photodynamic therapy of cancer: an update. CA Cancer J. Clin..

[6] Moan J, BERG K (1991). The photodegradation of porphyrins in cells can be used to estimate the lifetime of singlet oxygen. Photochem. Photobiol..

[7] Zheng, G. *The Porphyrin Handbook* **6**, 157–230 (Academic Press, San Diego, CA).

[8] Konan YN, Gurny R, Allémann E (2002). State of the art in the delivery of photosensitizers for photodynamic therapy. J. Photochem. Photobiol. B Biol..

[9] Ji Z (2006). Subcellular localization pattern of protoporphyrin IX is an important determinant for its photodynamic efficiency of human carcinoma and normal cell lines. J. Photochem. Photobiol. B Biol..

[10] Castano AP, Demidova TN, Hamblin MR (2004). Mechanisms in photodynamic therapy: part one—photosensitizers, photochemistry and cellular localization. Photo. Photodyn. Ther..

[11] Hsieh YJ, Wu CC, Chang CJ, Yu JS (2003). Subcellular localization of Photofrin® determines the death phenotype of human epidermoid carcinoma A431 cells triggered by photodynamic therapy: when plasma membranes are the main targets. J. Cell. Physiol..

[12] Neupert W, Herrmann JM (2007). Translocation of proteins into mitochondria. Annu. Rev. Biochem..

[13] Galluzzi L, Bravo-San Pedro JM, Kroemer G (2014). Organelle-specific initiation of cell death. Nat. Cell Biol..

[14] Deng J, Wang K, Wang M, Yu P, Mao L (2017). Mitochondria targeted nanoscale zeolitic imidazole framework-90 for ATP imaging in live cells. J. Am. Chem. Soc..

[15] Green, D. R. & Reed, J. C. Mitochondria and apoptosis. *Science* **281**, 1309–1312 (1998). 10.1126/science.281.5381.1309 9721092

[16] Poynton FE (2017). The development of ruthenium (ii) polypyridyl complexes and conjugates for in vitro cellular and in vivo applications. Chem. Soc. Rev..

[17] Pierroz V (2012). Molecular and cellular characterization of the biological effects of ruthenium (II) complexes incorporating 2-pyridyl-2-pyrimidine-4-carboxylic acid. J. Am. Chem. Soc..

[18] Zhou Zhixuan, Liu Jiangping, Rees Thomas W., Wang Heng, Li Xiaopeng, Chao Hui, Stang Peter J. (2018). Heterometallic Ru–Pt metallacycle for two-photon photodynamic therapy. Proceedings of the National Academy of Sciences.

[19] Tan Xu, Luo Shenglin, Long Lei, Wang Yu, Wang Dechun, Fang Shengtao, Ouyang Qin, Su Yongping, Cheng Tianmin, Shi Chunmeng (2017). Structure-Guided Design and Synthesis of a Mitochondria-Targeting Near-Infrared Fluorophore with Multimodal Therapeutic Activities. Advanced Materials.

[20] Jung HS (2015). Enhanced NIR radiation-triggered hyperthermia by mitochondrial targeting. J. Am. Chem. Soc..

[21] Zeng L (2017). The development of anticancer ruthenium (II) complexes: from single molecule compounds to nanomaterials. Chem. Soc. Rev..

[22] Smith AM, Mancini MC, Nie S (2009). Bioimaging: second window for in vivo imaging. Nat. Nanotechnol..

[23] Liu J (2015). Ruthenium (II) polypyridyl complexes as mitochondria-targeted two-photon photodynamic anticancer agents. Biomaterials.

[24] Zhang P (2015). Ruthenium (II) anthraquinone complexes as two-photon luminescent probes for cycling hypoxia imaging in vivo. Biomaterials.

[25] Chen Y (2016). Two-photon luminescent metal complexes for bioimaging and cancer phototherapy. Coord. Chem. Rev..

[26] He C, Lu K, Liu D, Lin W (2014). Nanoscale metal–organic frameworks for the co-delivery of cisplatin and pooled siRNAs to enhance therapeutic efficacy in drug-resistant ovarian cancer cells. J. Am. Chem. Soc..

[27] Furukawa H, Cordova KE, O’Keeffe M, Yaghi OM (2013). The chemistry and applications of metal-organic frameworks. Science.

[28] Morris W, Briley WE, Auyeung E, Cabezas MD, Mirkin CA (2014). Nucleic acid–metal organic framework (MOF) nanoparticle conjugates. J. Am. Chem. Soc..

[29] Levine DJ (2016). Olsalazine-based metal–organic frameworks as biocompatible platforms for H2 adsorption and drug delivery. J. Am. Chem. Soc..

[30] Lu K, He C, Lin W (2014). Nanoscale metal–organic framework for highly effective photodynamic therapy of resistant head and neck cancer. J. Am. Chem. Soc..

[31] Lu K (2016). Chlorin-based nanoscale metal–organic framework systemically rejects colorectal cancers via synergistic photodynamic therapy and checkpoint blockade immunotherapy. J. Am. Chem. Soc..

[32] Lan, G., Ni, K. & Lin, W. Nanoscale metal–organic frameworks for phototherapy of cancer. *Coord. Chem. Rev.*10.1016/j.ccr.2017.09.007 (2017). 10.1016/j.ccr.2017.09.007 PMC6366651 30739946

[33] Zheng X (2017). Metal–organic framework@ porous organic polymer nanocomposite for photodynamic therapy. Chem. Mater..

[34] Zeng Jin-Yue, Zou Mei-Zhen, Zhang Mingkang, Wang Xiao-Shuang, Zeng Xuan, Cong Hengjiang, Zhang Xian-Zheng (2018). π-Extended Benzoporphyrin-Based Metal–Organic Framework for Inhibition of Tumor Metastasis. ACS Nano.

[35] Lu K (2018). Low-dose X-ray radiotherapy–radiodynamic therapy via nanoscale metal–organic frameworks enhances checkpoint blockade immunotherapy. Nat. Biomed. Eng..

[36] Lan G (2017). Nanoscale metal–organic layers for deeply penetrating X‐ray‐induced photodynamic therapy. Angew. Chem..

[37] Ni K (2018). Nanoscale metal-organic frameworks enhance radiotherapy to potentiate checkpoint blockade immunotherapy. Nat. Commun..

[38] Chandel NS (2000). Reactive oxygen species generated at mitochondrial complex III stabilize hypoxia-inducible factor-1α during hypoxia a mechanism of O2 sensing. J. Biol. Chem..

[39] He C, Lu K, Lin W (2014). Nanoscale metal–organic frameworks for real-time intracellular pH sensing in live cells. J. Am. Chem. Soc..

[40] Ferrario A, von Tiehl K, Wong S, Luna M, Gomer CJ (2002). Cyclooxygenase-2 inhibitor treatment enhances photodynamic therapy-mediated tumor response. Cancer Res..

[41] Kessel D, Luo Y (1999). Photodynamic therapy: a mitochondrial inducer of apoptosis. Cell Death Differ..

[42] Moor AC (2000). Signaling pathways in cell death and survival after photodynamic therapy. J. Photochem. Photobiol. B Biol..

[43] Stockert JC, Blázquez-Castro A, Cañete M, Horobin RW, Villanueva Aacute (2012). MTT assay for cell viability: intracellular localization of the formazan product is in lipid droplets. Acta Histochem..

[44] Yousif LF, Stewart KM, Kelley SO (2009). Targeting mitochondria with organelle‐specific compounds: strategies and applications. Chembiochem.

[45] Omura T (1998). Mitochondria-targeting sequence, a multi-role sorting sequence recognized at all steps of protein import into mitochondria. J. Biochem..

[46] Agemy L (2011). Targeted nanoparticle enhanced proapoptotic peptide as potential therapy for glioblastoma. Proc. Natl. Acad. Sci. USA.

[47] Mileykovskaya E, Dowhan W (2000). Visualization of phospholipid domains in *Escherichia coli* by using the cardiolipin-specific fluorescent dye 10-N-nonyl acridine orange. J. Bacteriol..

[48] Marrache S, Dhar S (2012). Engineering of blended nanoparticle platform for delivery of mitochondria-acting therapeutics. Proc. Natl. Acad. Sci. USA.

[49] Smiley ST (1991). Intracellular heterogeneity in mitochondrial membrane potentials revealed by a J-aggregate-forming lipophilic cation JC-1. Proc. Natl. Acad. Sci. USA.

[50] Zielonka J (2017). Mitochondria-targeted triphenylphosphonium-based compounds: syntheses, mechanisms of action, and therapeutic and diagnostic applications. Chem. Rev..

[51] Marrache S, Tundup S, Harn DA, Dhar S (2013). Ex vivo programming of dendritic cells by mitochondria-targeted nanoparticles to produce interferon-gamma for cancer immunotherapy. ACS Nano.

[52] Marrache S, Pathak RK, Dhar S (2014). Detouring of cisplatin to access mitochondrial genome for overcoming resistance. Proc. Natl. Acad. Sci. USA.

[53] Zhou Z, Song J, Nie L, Chen X (2016). Reactive oxygen species generating systems meeting challenges of photodynamic cancer therapy. Chem. Soc. Rev..

[54] Franken NA, Rodermond HM, Stap J, Haveman J, Van Bree C (2006). Clonogenic assay of cells in vitro. Nat. Protoc..

[55] Bolte S, Cordelieres F (2006). A guided tour into subcellular colocalization analysis in light microscopy. J. Microsc..

