# Supplementary material for: Nanoscale metal-organic frameworks for mitochondria-targeted radiotherapy-radiodynamic therapy
Source: Nat Commun. 2018 Oct 17;9:4321. doi: 10.1038/s41467-018-06655-7 (PMC6193046; doi:10.1038/s41467-018-06655-7)
Supplement: Supplementary file 1 — Supplementary Information [file 41467_2018_6655_MOESM1_ESM.pdf]

Supplementary Information

Nanoscale Metal-Organic Frameworks for Mitochondria-Targeted Radiotherapy-Radiodynamic Therapy

Kaiyuan Ni[1,†], Guangxu Lan[1,†], Samuel S. Veroneau[1], Xiaopin Duan[1], Yang Song[1], Wenbin Lin[1,2*]

[1]Department of Chemistry and [2]Department of Radiation and Cellular Oncology and Ludwig Center for Metastasis Research, The University of Chicago, Chicago, IL 60637, USA.

[*]Corresponding author. E-mail: wenbinlin@uchicago.edu

[†]These authors contributed equally to this work.

Supplementary Methods

H_2DBB-Ru Synthesis

Dimethyl 2,2'-bipyridine-5,5'- dibenzoate (320 mg, 750 μmol) and Ru(bpy)$_2$Cl$_2$ (350 mg, 725 μmol) were suspended in 10 mL EtOH and 10 mL $CHCl_3$ under nitrogen. The reaction mixture was heated to 110 °C for 72 h. The solution was then cooled to room temperature, filtered, and concentrated in vacuo. Diethyl ether (30 mL) was added to the concentrated solution to afford the product as a red precipitate (Me$_2$DBB-Ru). The Me$_2$DBB-Ru (300 mg) from above was suspended in the mixture of 20 mL THF, 20 mL methanol and 10 mL 1.8 M NaOH/H_2O solution. The reaction mixture was heated to reflux overnight. 12 M HCl was added to the suspension to tune the pH to 1. The red solid product (H_2DBB-Ru) was isolated by vacuum filtration and washed with THF, methanol and ether. ^{1}H-NMR (500 HZ, DMSO-d6): δ=9.01 (d, 2H), 8.85 (m, 4H), 8.56 (m, 2H), 8.24 (m, 2H), 8.21 (m, 2H), 7.96 (m, 6H), 7.85 (d, 2H), 7.78 (d, 2H), 7.58 (m, 8H).

X-ray Absorption Spectroscopy

Data Collection X-ray absorption data were collected at Beamline 10-BM-A, B at the Advanced Photon Source (APS) at Argonne National Laboratory. Spectra were collected at the ruthenium K-edge (22117 eV) in transmission mode. The X-ray beam was monochromatized by a Si(111) monochromater and detuned by 50% to reduce the contribution of higher-order harmonics below the level of noise. A metallic ruthenium foil standard was used as a reference for energy calibration and was measured simultaneously with experimental samples. The incident beam intensity (I_0), transmitted beam intensity (I_t), and reference (I_r) were measured by 20 cm ionization chambers with gas compositions of 44% N_2 and 56% Ar, 5% N_2 and 95% Ar, and 100% N_2, respectively. Data were collected over six regions: -250 to -30 eV (10 eV step size, dwell time of 0.25 s), -30 to -12 eV (5 eV step size, dwell time of 0.5 s), -12 to 30 eV (1.1 eV step size, dwell time of 1 s), 30 eV to 6 Å^{-1}, (0.05 Å^{-1} step size, dwell time of 2 s), 6 Å^{-1} to 12 Å^{-1}, (0.05 Å^{-1} step size, dwell time of 4 s), 12 Å^{-1} to 15 Å^{-1}, (0.05 Å^{-1} step size, dwell time of 8 s). Multiple X-ray absorption spectra were collected at room temperature for each sample. Samples were ground and mixed with polyethyleneglycol (PEG) and packed in a 6-shooter sample holder to achieve adequate absorption length.

Data Processing Data was processed using the Athena and Artemis programs of the IFEFFIT package based on FEFF 6. Prior to merging, spectra were calibrated against the reference spectra and aligned to the first peak in the smoothed first derivative of the absorption spectrum, the background noise was removed, and the spectra were processed to obtain a normalized unit edge step.

EXAFS Fitting Fits of the EXAFS region were performed using the Artemis program

of the IFEFFIT package. Fits were performed in R space, with a *k*-weight of 3 for Ru samples. Refinement was performed by optimizing an amplitude factor S_0^2 and energy shift ΔE_0 which are common to all paths, in addition to parameters for bond length (ΔR) and Debye-Waller factor (σ^2). The fitting models for H_2DBB-Ru and Hf-DBB-Ru were based on the crystal structure OPELOL obtained from the Cambridge Crystallographic Database.

Hydroxyl radical produced by Fenton reaction

Hf-DBA, and Hf-DBB-Ru were suspended in water at equivalent Hf concentrations of 20 μM. Aqueous solutions of 5 μM APF, 50 μM $FeCl_2$, and various H_2O_2 concentration of 0, 400, 800, 1600, 3200, and 6400 μM were separately prepared. 100 μL of nanoparticle suspension and aqueous solution of APF, $FeCl_2$, and H_2O_2 were added to a 96-well plate and the fluorescence signals were immediately collected with a Xenogen IVIS 200 imaging system (Xenogen, USA). Nanoparticle-free aqueous solutions of APF, $FeCl_2$, and H_2O_2 served as controls.

Cellular uptake

The cellular uptake of Hf-DBA or Hf-DBB-Ru was evaluated in MC38 cells. Cells were seeded on 6-well plate at 1×10^6/well and further cultured for 12 h. Particles were added to the cells at a Hf concentration of 20 μM. After incubation of 1, 2, 4 and 8 hours, the cells were collected and the cell numbers were counted by a hemocytometer. Cells were washed with PBS at least three times and then digested with 1% hydrofluoric acid and concentrated nitric acid in a microwave reactor and the Hf concentrations were determined by ICP-MS. Results were expressed as the amount of Hf (nmol) per 10^5 cells.

For the comparison of cellular uptake of Hf-DBA and Hf-DBA-R, MC38 cells were seeded on 6-well plate at 1×10^6/well and further cultured for 12 h. Hf-DBA nMOFs with or without rhodamine labeling were added to the cells at a Hf concentration of 20 μM. After incubation of 4 hours, the cells were collected and the numbers of cell were counted by a hemocytometer. Cells were washed with PBS for at least three times then digested with 1% hydrofluoric acid and concentrated nitric acid in a microwave reactor (CEM, USA) and the Hf concentrations were determined by ICP-MS (Agilent, USA). Results were expressed as the amount of Hf (ng) per 10^5 cells.

Time-dependent Co-localization

MC38 cells were cultured in 35 mm tissue culture dishes overnight and incubated with Hf-DBA-R or Hf-DBB-Ru at an equivalent dose of 20 μM for 1, 2, 4, and 8 h. Cellular nuclei and mitochondria were labeled with hoechest 33258 and rhodamine 123, respectively. After washing with PBS, treated MC38 cells at each time point were then co-stained with Hoechest 33342 and Rhodamine 123 for 10 min at room temperature. Cells were then fixed with 4% paraformaldehyde at 4 °C for 30 min. The

slides were then washed with PBS and observed under CLSM. Pearson' s coefficient was quantified using the Co-localization Analysis plugin for ImageJ.

Morphological Change Observation

MC38 cells seeded on cover slides in 35 mm tissue culture dishes overnight. Hf-DBA, H_2DBB-Ru or Hf-DBB-Ru was added to the cells at an equivalent dose of 20 μM. Cells incubated with PBS served as a control. After incubation of 4 hours, cells were irradiated upon X-ray irradiation (250 kVp, 15 mA, 1 mm Cu filter) at a dose of 0 or 2 Gy. After 72-hour incubation, slides were washed with PBS and directly observed and the bright field images were obtained using a cutoff filter transmitting light >590 nm.

Respiratory Chain Activity Assay

Respiratory chain activity was assayed with (3-(4,5-Dimethylthiazol-2-yl)-2,5-di-phenyltetrazolium bromide (MTT). MC38 cells seeded on cover slides in in 35 mm tissue culture dishes overnight. Hf-DBA, H_2DBB-Ru or Hf-DBB-Ru was added to the cells at an equivalent dose of 20 μM. Cells incubated with PBS served as a control. After incubation of 4 hours, cells were irradiated upon X-ray irradiation (250 kVp, 15 mA, 1 mm Cu filter) at a dose of 0 or 2 Gy. After 24-hour incubation, MTT was added with concentration of 0.1 mg/mL and stained for 20 min at room temperature. The slides were then washed with PBS and observed under the stereomicroscope.

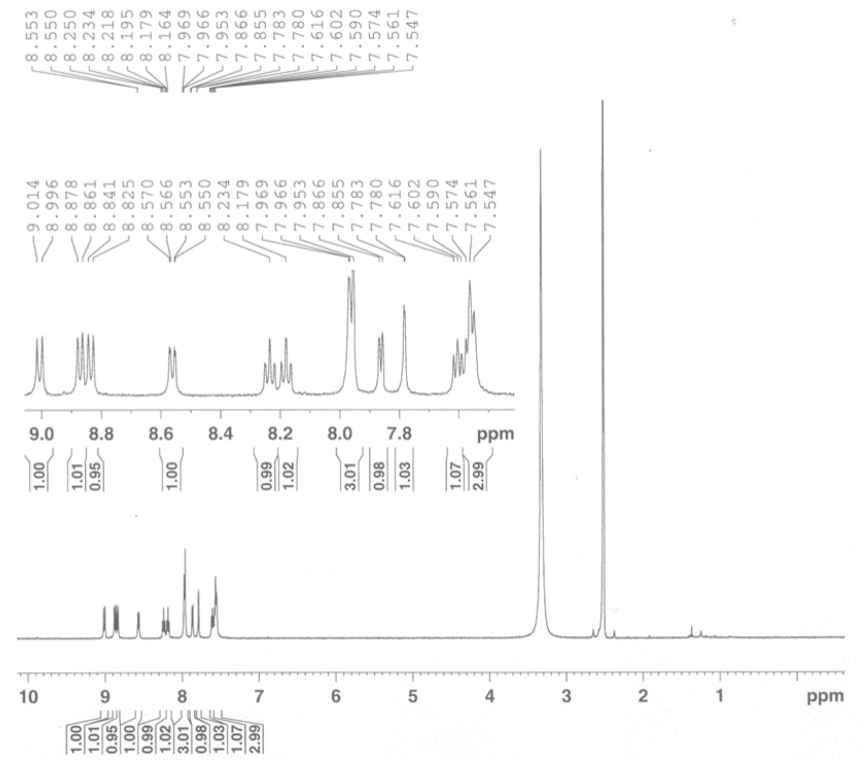

Supplementary Figure 1 NMR of H_2DBB-Ru. One of more than five repetitions with similar results is shown.

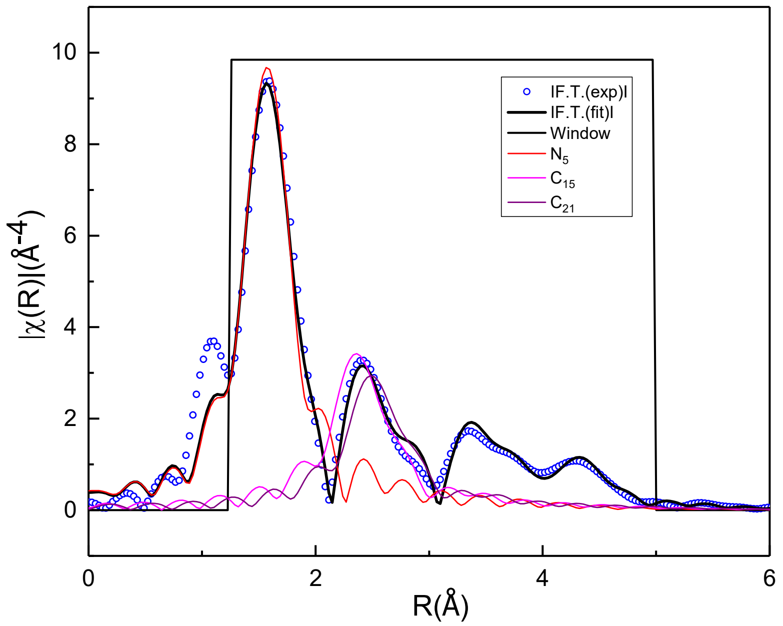

Supplementary Figure 2 EXFAS spectra (blue circles) and fits (black solid line) in R-space at the Ru K-edge adsorption of H_2DBB-Ru. The result was obtained without repetition.

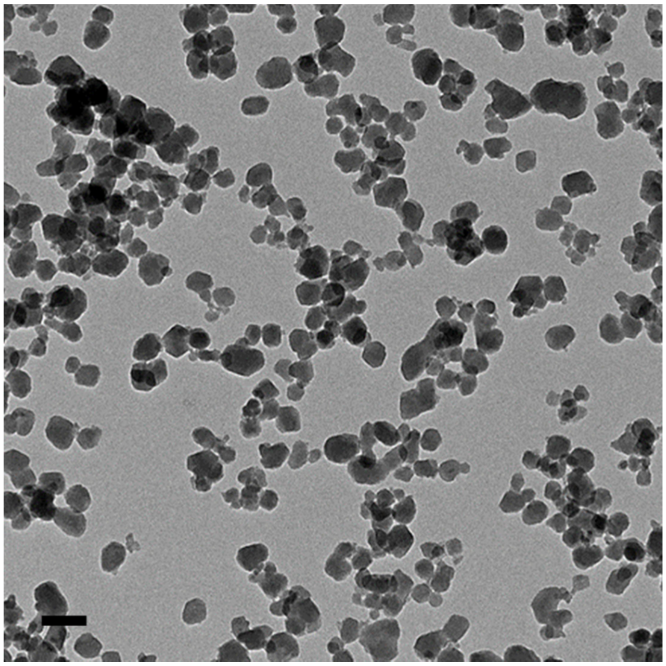

Supplementary Figure 3 TEM image of Hf-DBA. Scale bar = 100 nm. One of more than five repetitions with similar results is shown.

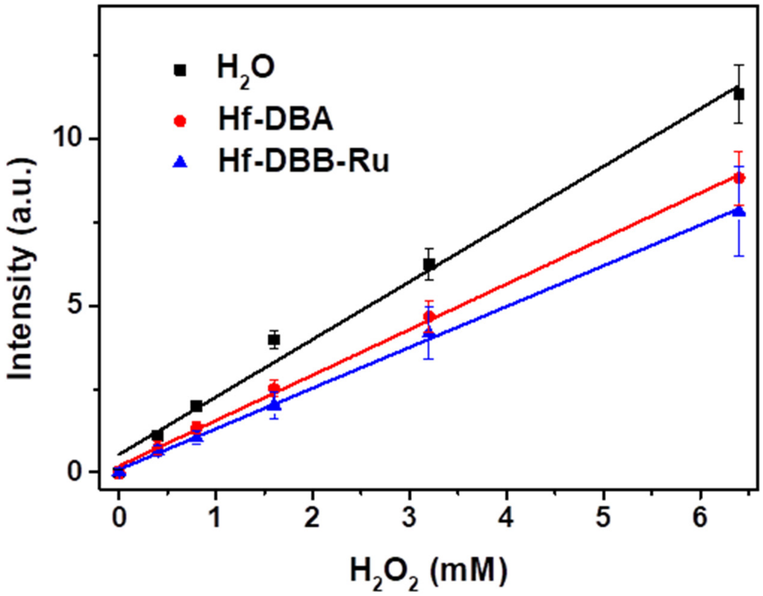

Supplementary Figure 4 APF fluorescence of H_2O, Hf-DBA, and Hf-DBB-Ru with Fenton reaction at equivalent Hf concentrations of 20 µM, n = 6. The ratio of fitting slope of Hf-DBA or Hf-DBB-Ru to H_2O is 0.69 or 0.60, respectively. The error bars represent s.d. values. The result was obtained without repetition.

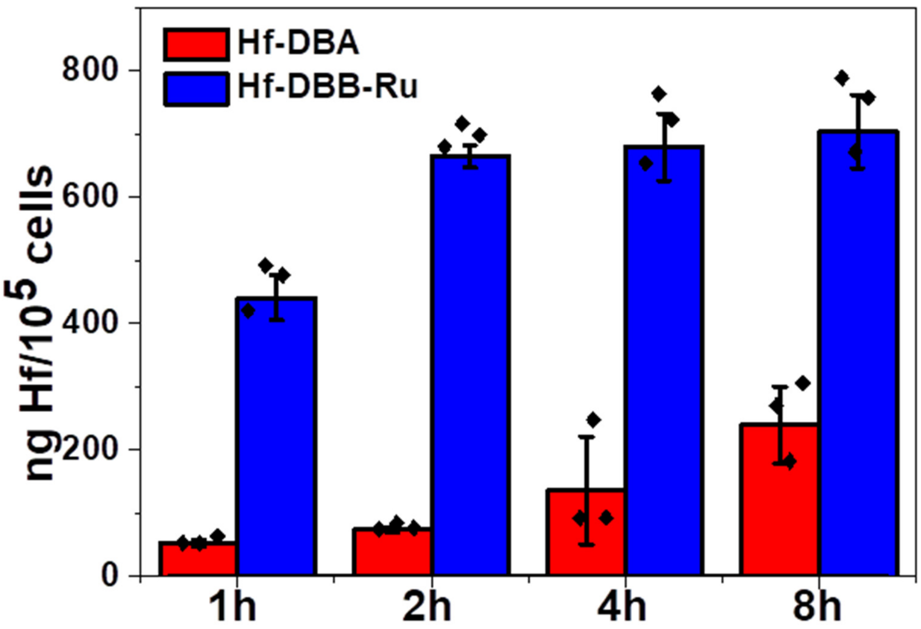

Supplementary Figure 5 Cellular uptake of Hf-DBA or Hf-DBB-Ru after 1, 2, 4 or 8 hour incubation with equivalent Hf concentrations of 20 μM. The Hf concentrations were determined by ICP-MS, n = 3. The dots and error bars represent individual data points and s.d. values. The result was obtained without repetition.

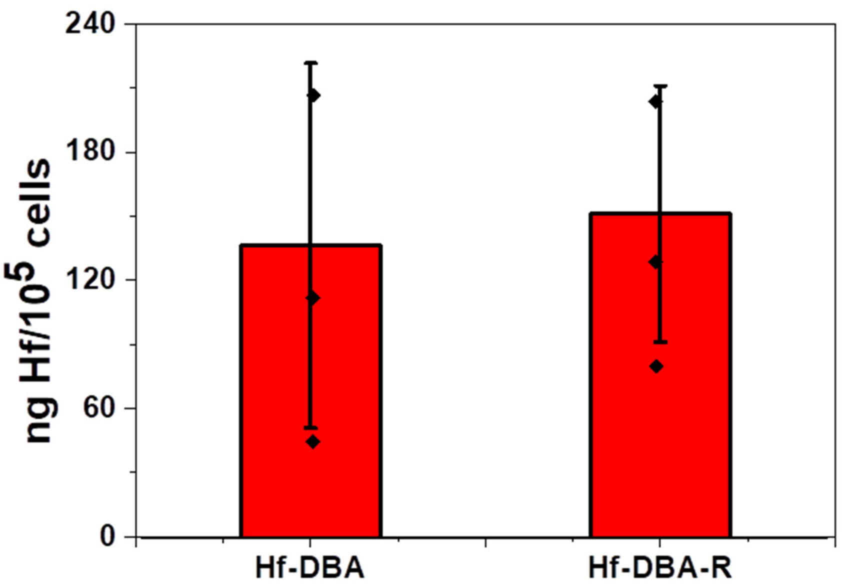

Supplementary Figure 6 Cellular uptake of Hf-DBA and Hf-DBA-R after 4 hour incubation with equivalent Hf concentrations of 20 μM. The Hf concentrations were determined by ICP-MS, n = 3. The dots and error bars represent individual data points and s.d. values. The result was obtained without repetition.

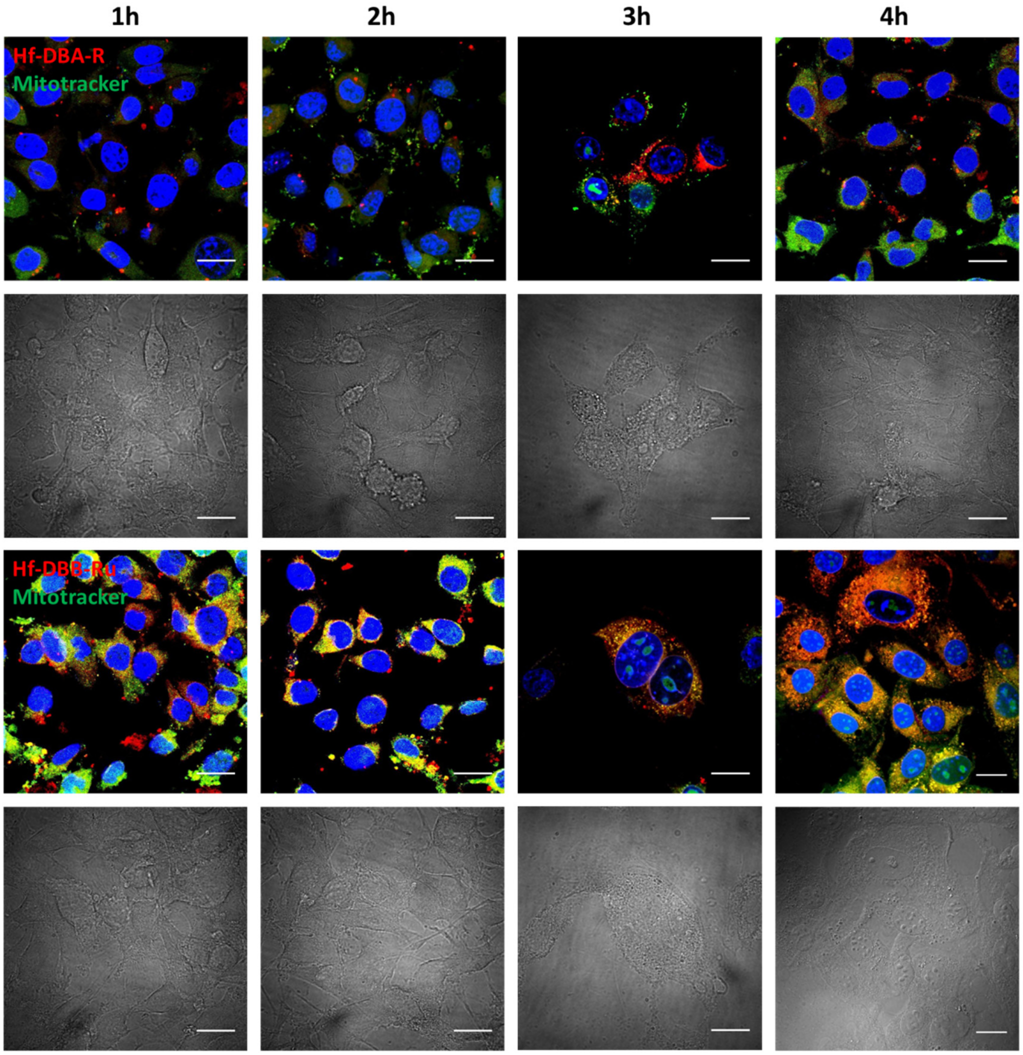

Supplementary Figure 7 Co-localization of Hf-DBA-R or Hf-DBB-Ru with mitochondria after different nMOFs incubation time periods. The cells were first treated with Hf-DBA-R or Hf-DBB-Ru for 1, 2, 4 or 8 hour incubation with equivalent Hf concentrations of 20 μM. After washing with PBS, treated MC38 cells at each time point were then co-stained with commercial organelle trackers for 10 min at room temperature. Blue: Hoechst 33342; Green: Rhodamine 123; Red: Hf-DBA-R or Hf-DBB-Ru. From top to bottom: Hf-DBA-R and corresponding bright field images; Hf-DBB-Ru and corresponding bright field images. From left to right: 1h, 2h, 4h, and 8h, respectively. Scale bar = 20 μm. The images were obtained with two repetitions to afford similar results.

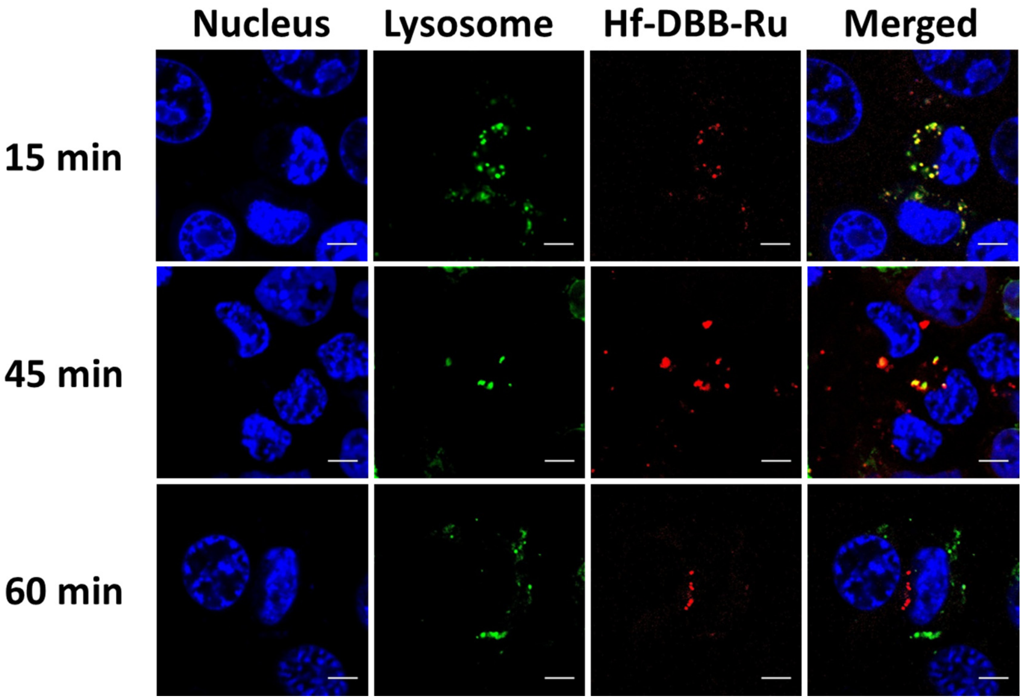

Supplementary Figure 8 Endocytosis and endosomal escape of Hf-DBB-Ru by time-dependent confocal microscope imaging. The cells were incubated with Hf-DBB-Ru at equivalent Hf concentrations of 20 μM for 15 to 60 minutes. After washing with PBS, treated MC38 cells at each time point were co-stained with commercial organelle trackers for 10 min at room temperature. Blue: Hoechst 33342 for nuclei; Green: Lysotracker Green; Red: Hf-DBB-Ru. Scale bar = 5 μm. The result was obtained without repetition.

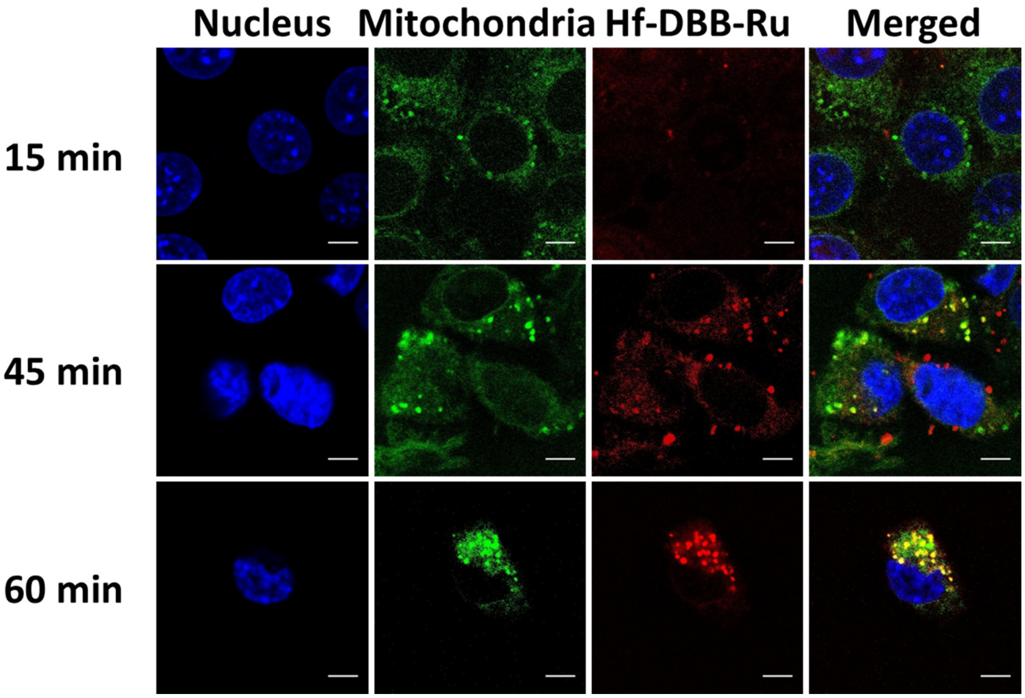

Supplementary Figure 9 Time-dependent mitochondrial enrichment of Hf-DBB-Ru by confocal microscope imaging. The cells were incubated with Hf-DBB-Ru at equivalent Hf concentrations of 20 μM for 15 to 60 minutes. After washing with PBS, treated MC38 cells at each time point were co-stained with commercial organelle trackers for 10 min at room temperature. Blue: Hoechst 33342 for nuclei; Green: Rhodamine 123 for mitochondria; Red: Hf-DBB-Ru. Scale bar = 5 μm. The result was obtained without repetition.

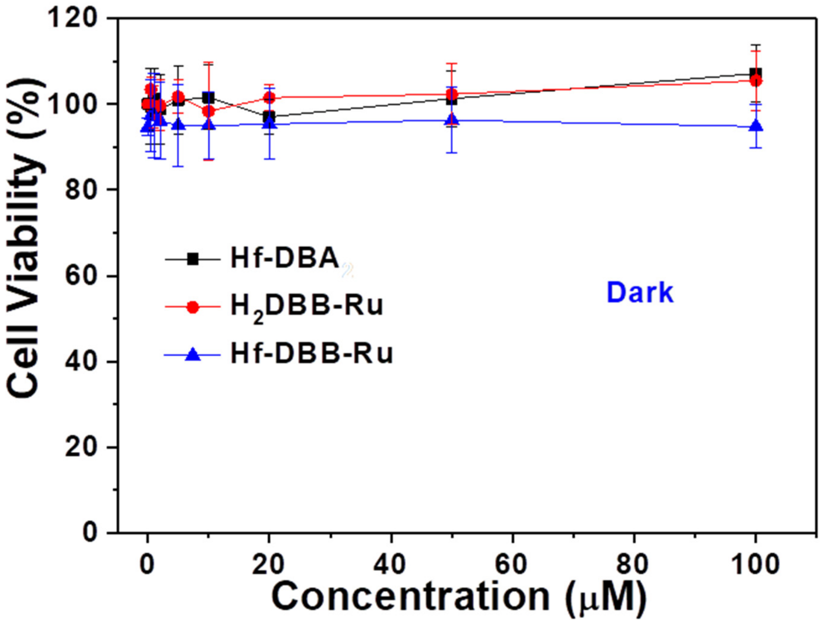

Supplementary Figure 10 Dark cytotoxicity of Hf-DBA, H_2DBB-Ru, or Hf-DBB-Ru against MC38 cells at different concentrations, n = 6. The error bars represent s.d. values. One of two repetitions with similar results is shown.

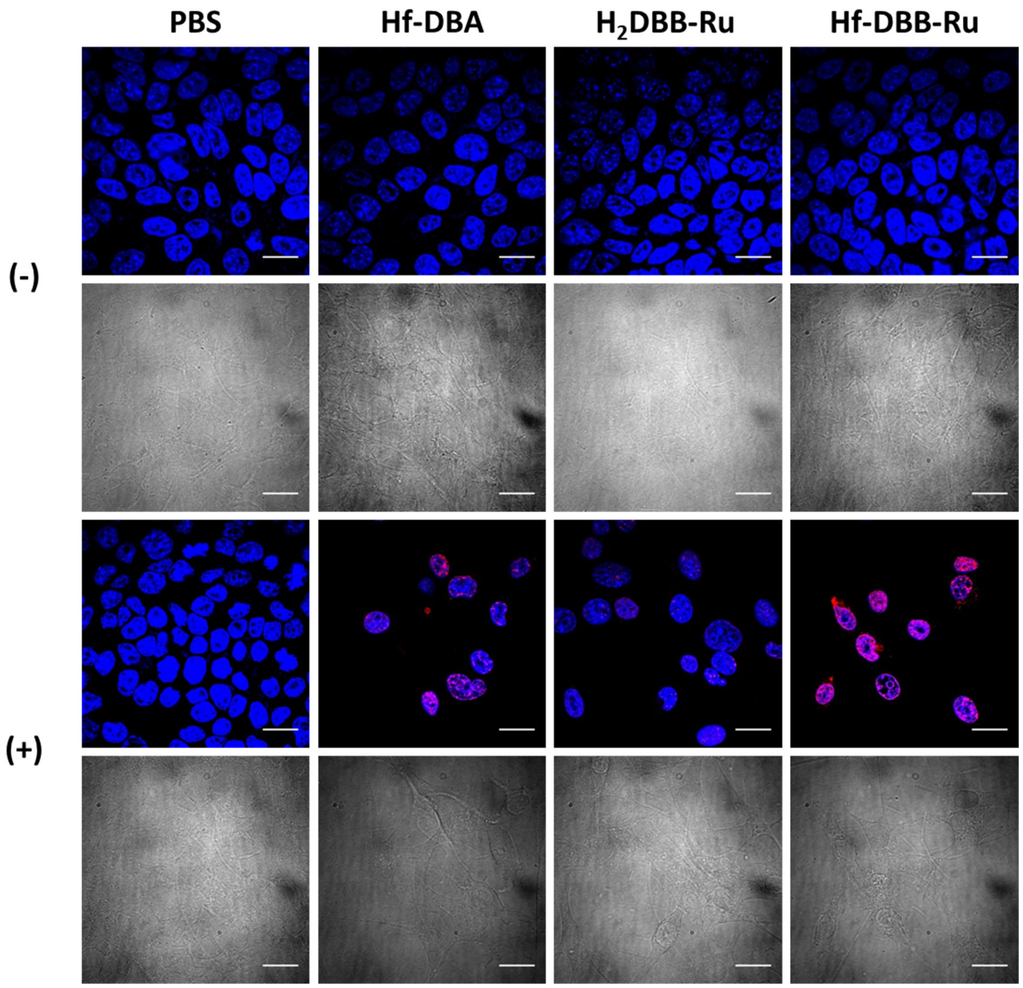

Supplementary Figure 11 Representative γ-H2AX immunostaining assay showing the DNA double strain breaks (DSBs) in MC38 cells. Cells were treated with PBS, Hf-DBA radiosensitizers, H_2DBB-Ru or Hf-DBB-Ru, with (+) or without (-) X-ray irradiation. Blue and red fluorescence show DAPI-stained nucleus and antibody-labeled γ-H2AX in the cells, respectively. From left to right: PBS, Hf-DBA, H_2DBB-Ru or Hf-DBB-Ru, respectively. Scale bar = 20 μm. One of two repetitions with similar results is shown.

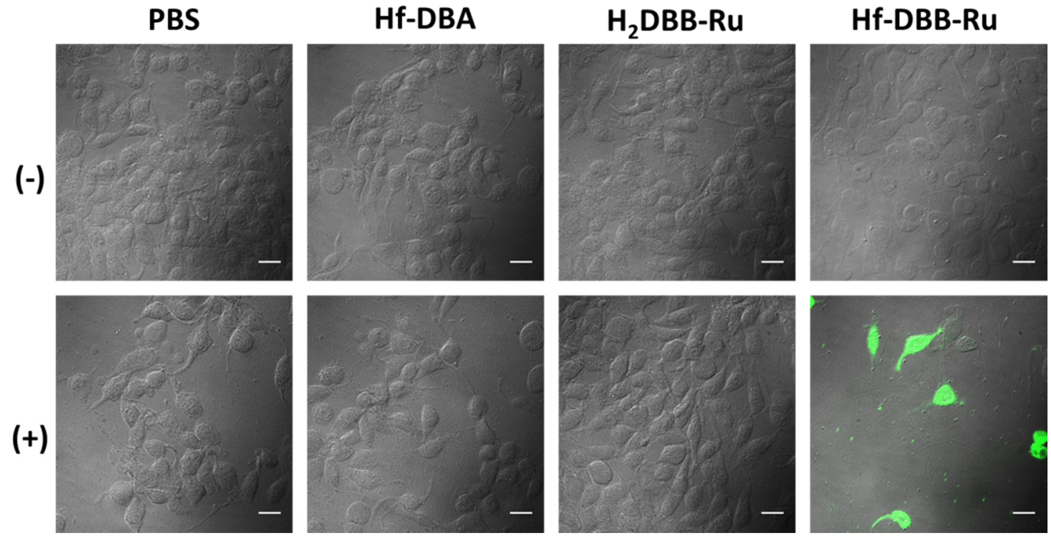

Supplementary Figure 12 Intracellular $^1O_2$ generation detected by SOSG. MC38 cells were preloaded with SOSG, incubated with PBS, Hf-DBA, H_2DBB-Ru, or Hf-DBB-Ru and irradiated at 0 (-) or 2 (+) Gy. Green fluorescence from SOSG captured $^1O_2$. From left to right: PBS control, Hf-DBA, H_2DBB-Ru, or Hf-DBB-Ru, respectively. Scale bar = 20 μm. One of two repetitions with similar results is shown.

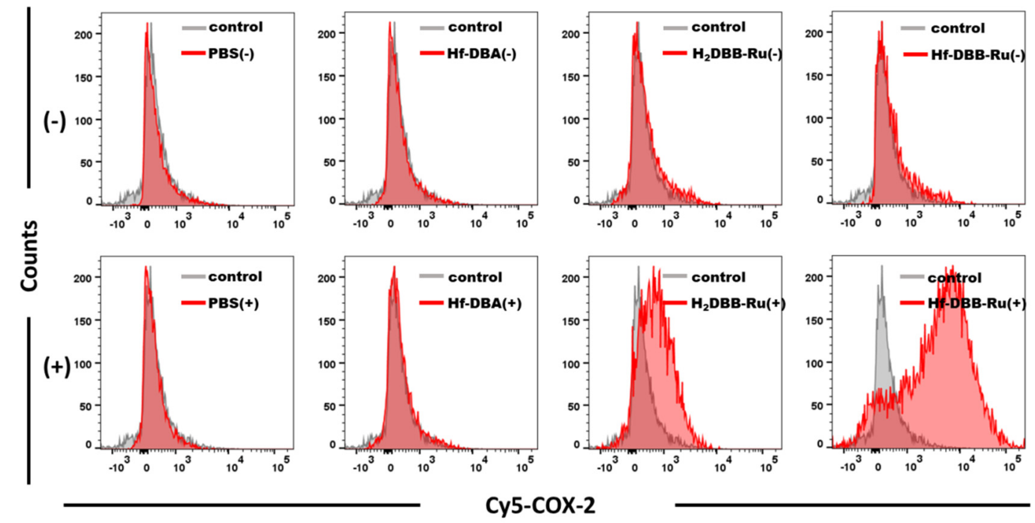

Supplementary Figure 13 Quantification of COX-2 by flow cytometry after cells incubated with PBS, Hf-DBA, H_2DBB-Ru, or Hf-DBB-Ru and irradiated with X-rays at a dose of 0 (-) or 2 (+) Gy. From left to right: PBS control, Hf-DBA, H_2DBB-Ru, or Hf-DBB-Ru, respectively. The results were obtained with one repetition to afford similar results.

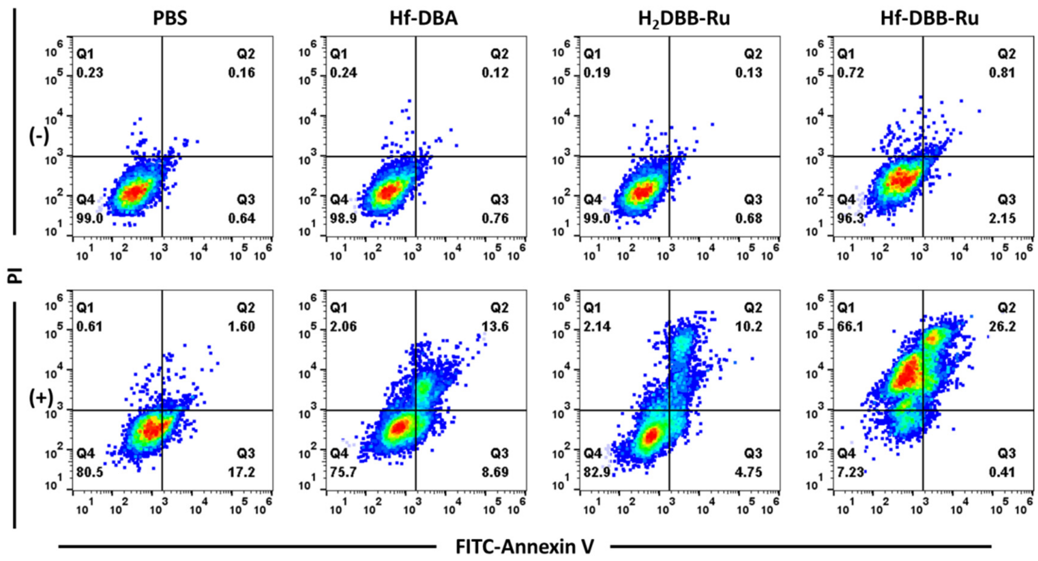

Supplementary Figure 14 Annexin V/PI analysis of MC38 cells. Cells were incubated with PBS, Hf-DBA, H_2DBB-Ru, or Hf-DBB-Ru and irradiated with X-rays at a dose of 0 (-) or 2 (+) Gy. The quadrants from lower left to upper left (counter clockwise) represent healthy, early apoptotic, late apoptotic, and necrotic cells, respectively. The percentage of cells in each quadrant was shown on the graphs. The results were obtained with one repetition to afford similar results.

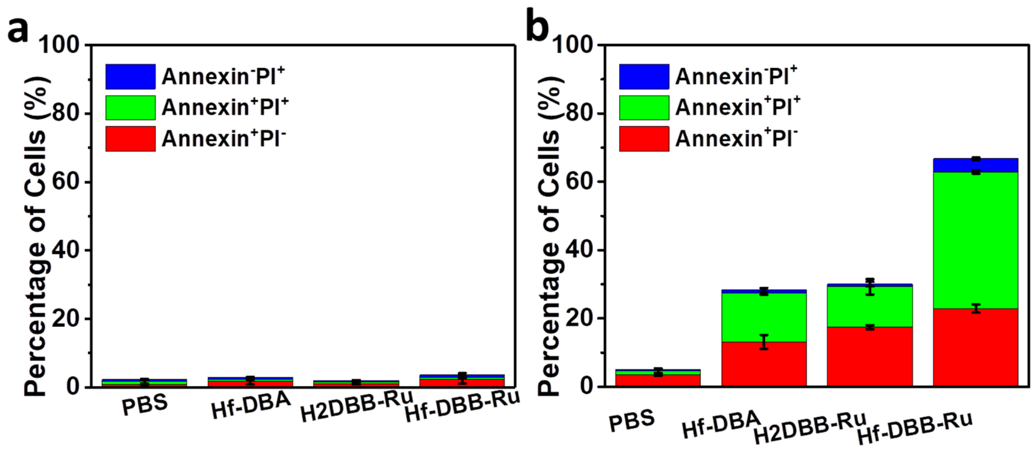

Supplementary Figure 15 Statistical analysis of the percentages of early apoptotic cell (red brick, Annexin$^+$PI$^-$), late apoptotic cell (green brick, Annexin$^+$PI$^+$), or necrotic cell (blue brick, Annexin$^-$PI$^+$) 48 hours after treated with PBS, Hf-DBA, H_2DBB-Ru, or Hf-DBB-Ru at an equivalent dose of 20 μM without (a) or with (b) X-ray irradiation, n = 3. From left to right: PBS control, Hf-DBA, H_2DBB-Ru, or Hf-DBB-Ru, respectively. The error bars represent s.d. values.

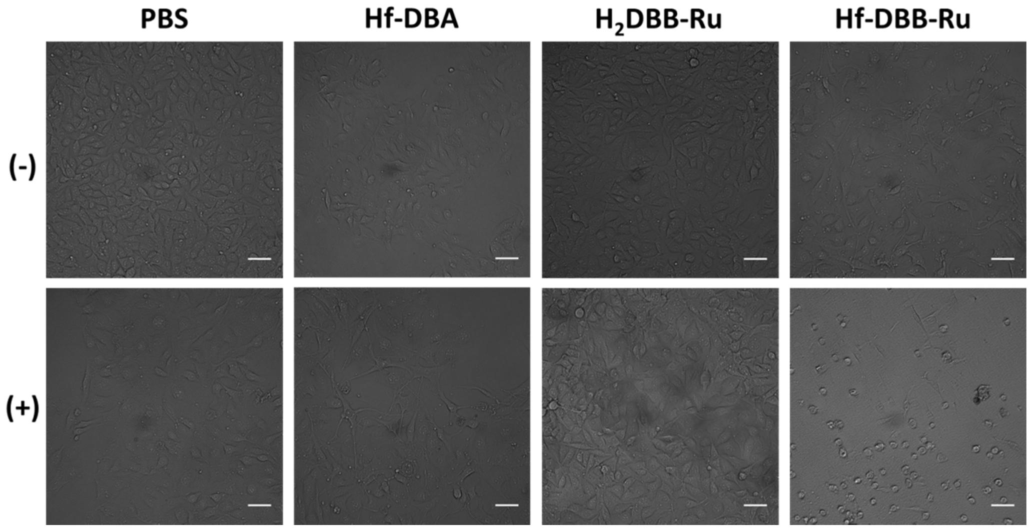

Supplementary Figure 16 Representative optical images of MC38 cells show cell morphological changes. 72 hours after treated with PBS, Hf-DBA, H_2DBB-Ru, or Hf-DBB-Ru at an equivalent dose of 20 μM upon X-ray irradiation at 0 (-) or 2 (+) Gy dose, cells were scanned to show the cell morphological changes. From left to right: PBS control, H_2DBB-Ru, Hf-DBA, or Hf-DBB-Ru, respectively. Scale bar = 50 μm. The images were obtained with one repetition to afford similar results.

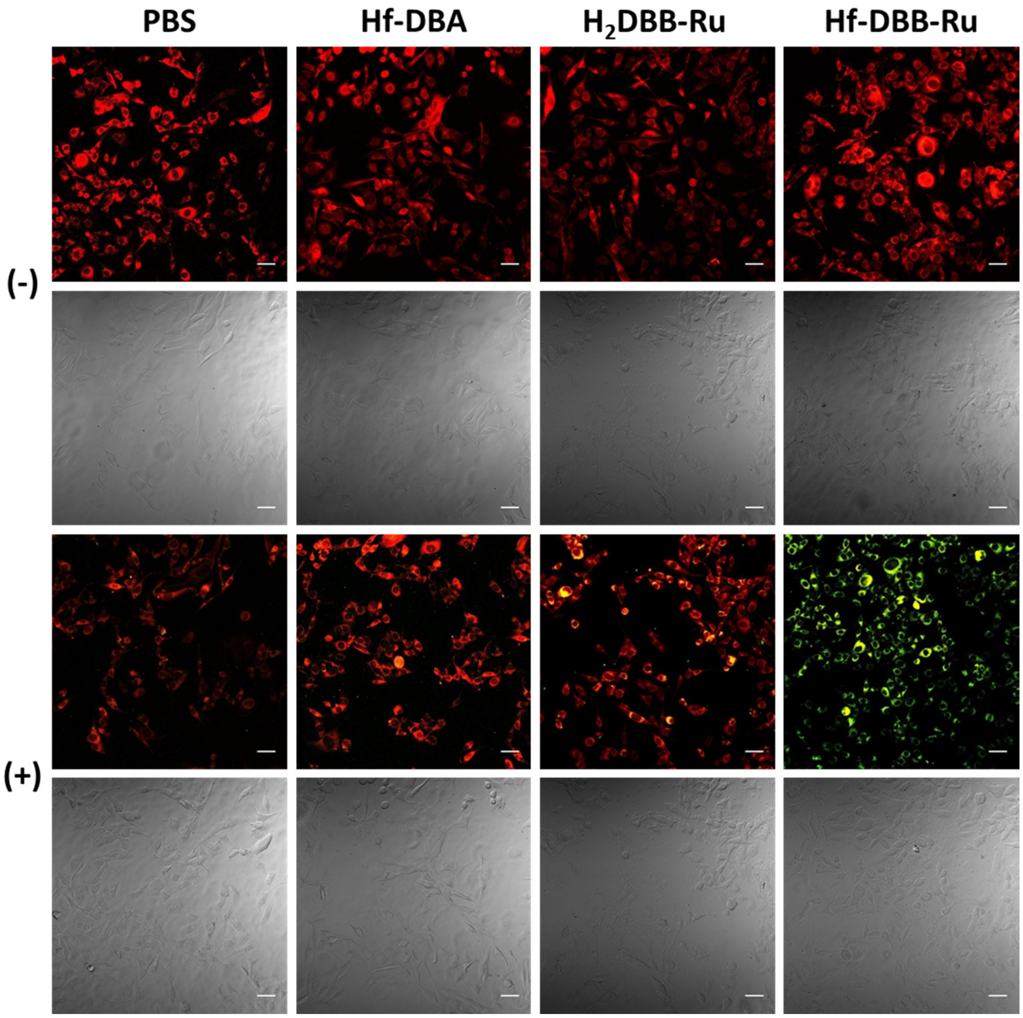

Supplementary Figure 17 JC-1 staining for detecting mitochondria membrane depolarization. MC38 cells treated with PBS, Hf-DBA, H_2DBB-Ru, or Hf-DBB-Ru at an equivalent dose of 20 μM upon X-ray irradiation at 0 (-) or 2 (+) Gy dose were stained with JC-1 4 h after irradiation. Green fluorescence indicates the monomerization of JC-1, suggesting the decrease of mitochondria membrane potential. From top to bottom: dark control and corresponding bright field images; X-ray irradiated groups and corresponding bright field images. From left to right: PBS control, Hf-DBA, H_2DBB-Ru, or Hf-DBB-Ru, respectively. Scale bar = 50 μm. The images were obtained with one repetition to afford similar results.

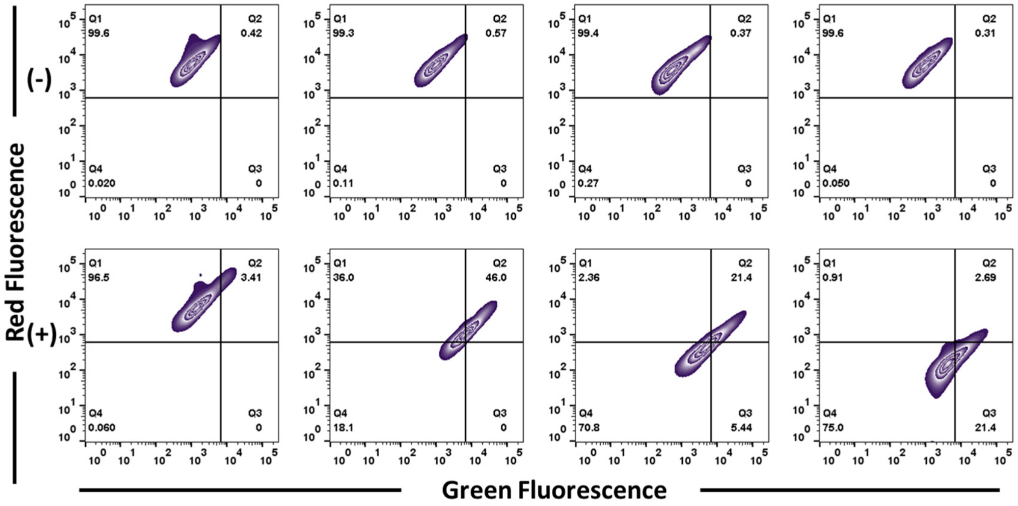

Supplementary Figure 18 JC-1 staining for detecting mitochondria membrane depolarization by flow cytometry analysis. MC38 cells treated with PBS, Hf-DBA, H_2DBB-Ru, or Hf-DBB-Ru at an equivalent dose of 20 μM upon X-ray irradiation at 0 (-) or 2 (+) Gy dose were stained with JC-1 4 h after irradiation. Green fluorescence indicates the monomerization of JC-1, suggesting the decrease of mitochondria membrane potential. From left to right: PBS control, Hf-DBA, H_2DBB-Ru, or Hf-DBB-Ru, respectively. The flow cytometry result was obtained with one repetition to afford similar results.

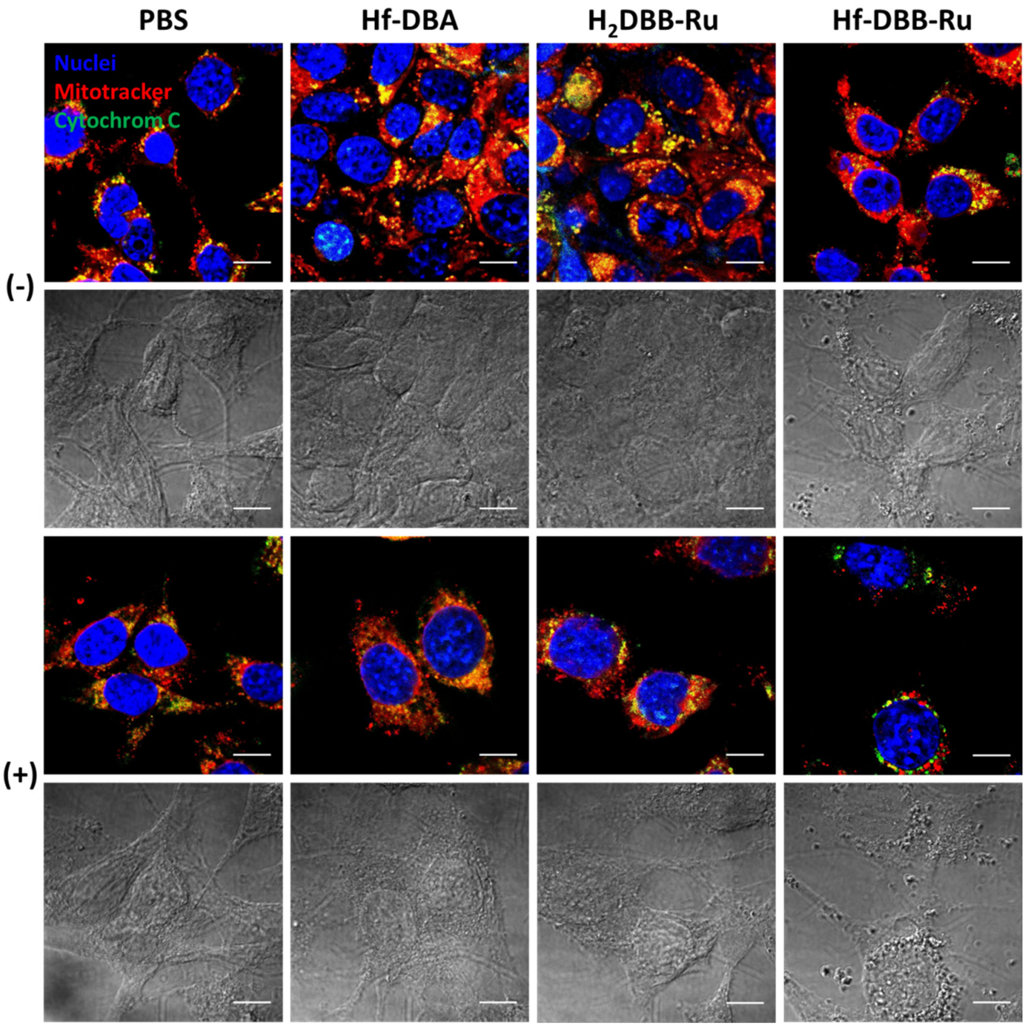

Supplementary Figure 19 Cytochorome c staining for presenting the release of cytochorome c from damaged mitochondria. MC38 cells were treated with PBS, Hf-DBA, H_2DBB-Ru, or Hf-DBB-Ru at an equivalent dose of 20 μM upon X-ray irradiation at 0 (-) or 2 (+) Gy dose. Cells were then stained with Mitotracker Red, fixed and permeabilized 8 h after irradiation. After stained with FITC-conjugated cytochrome c antibody and DAPI, slides were observed under CLSM. The deco-localization of green and red fluorescence indicates the release of cytochrome c from mitochondria. From top to bottom: dark control and corresponding bright field images; X-ray irradiated groups and corresponding bright field images. From left to right: PBS control, Hf-DBA, H_2DBB-Ru, or Hf-DBB-Ru, respectively. Scale bar = 10 μm. The images were obtained with one repetition to afford similar results.

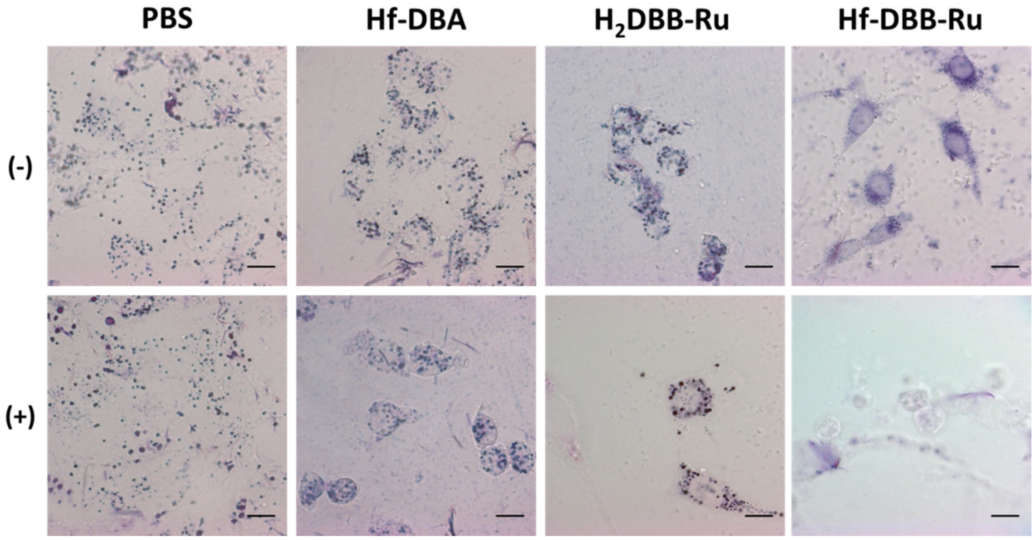

Supplementary Figure 20 Viability test for intact mitochondria and respiratory activity. MC38 cells were treated with after treated with PBS, Hf-DBA, H_2DBB-Ru, or Hf-DBB-Ru at an equivalent dose of 20 μM upon X-ray irradiation at 0 (-) or 2 (+) Gy dose. After incubation for 24 h, MTT was added to the cells for 20 mins to measure respiratory activity as the amount of MTT reduced to formazan. Dark blue "grains" in the image correspond to generated formazan microgranules. From left to right: PBS, Hf-DBA, H_2DBB-Ru, or Hf-DBB-Ru, respectively. Scale bar = 10 μm. The images were obtained with one repetition to afford similar results.

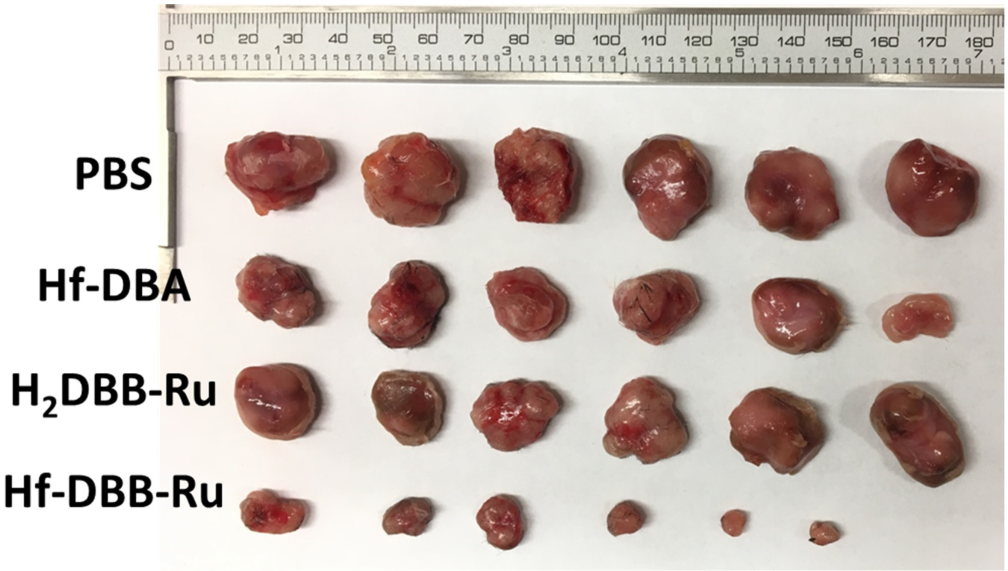

Supplementary Figure 21 Photos of excised tumors of the MC38 tumor model with different i.t. injected treatments, n = 6. From top to bottom: PBS, Hf-DBA, H_2DBB-Ru, and Hf-DBB-Ru, respectively. The result was obtained without repetition.

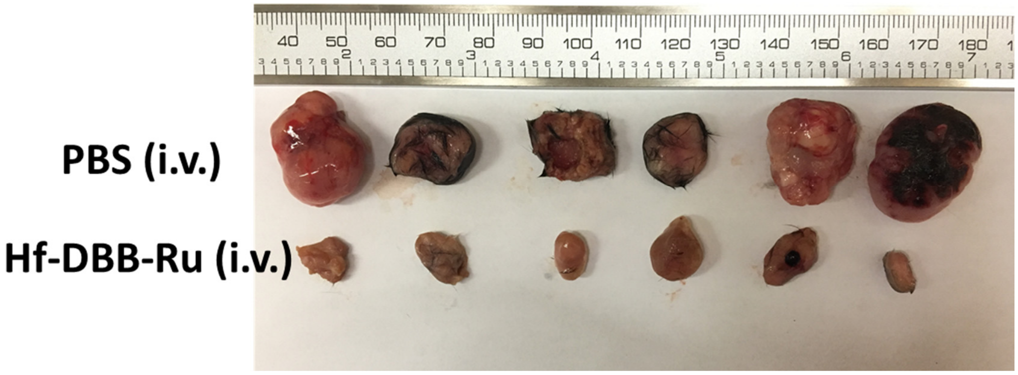

Supplementary Figure 22 Photos of excised tumors of the MC38 model i.v. injected with PBS or Hf-DBB-Ru followed by X-ray irradiation, n = 6. The result was obtained without repetition.

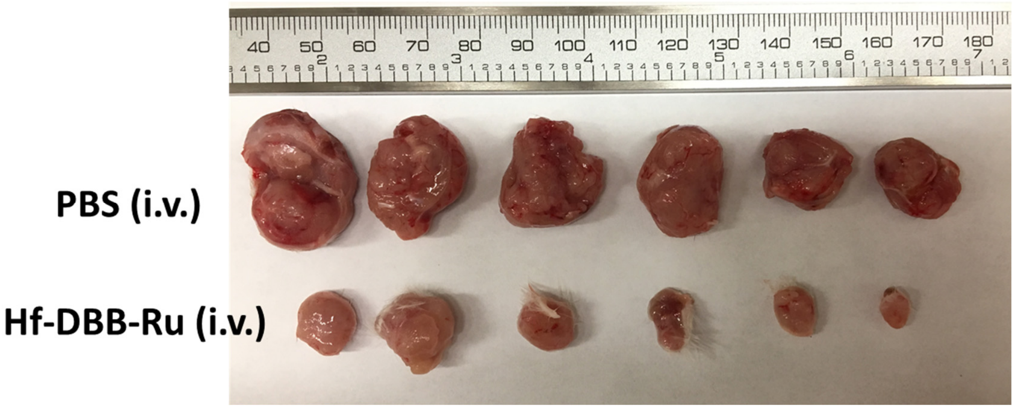

Supplementary Figure 23 Photos of excised tumors of the CT26 tumor model i.v. injected with PBS or Hf-DBB-Ru followed by X-ray irradiation, n = 6. The result was obtained without repetition.

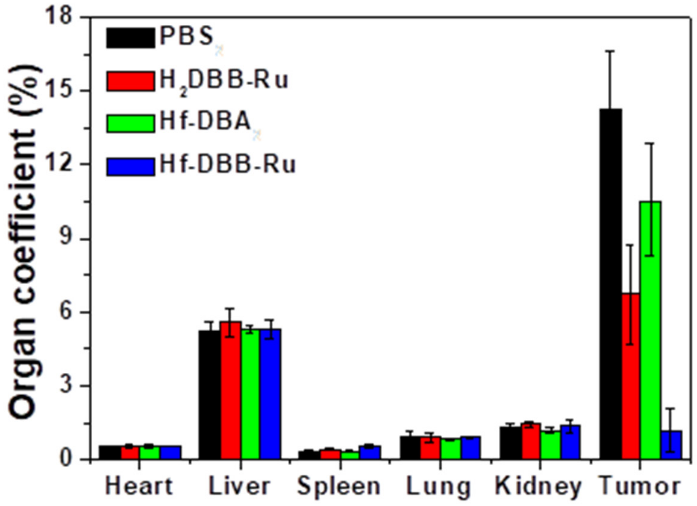

Supplementary Figure 24 Comparison of organ coefficients. MC38 tumor-bearing mice treated with i.t. injected PBS, Hf-DBA, DBB-Ru, or Hf-DBB-Ru followed by X-ray irradiation were euthanized and majors organs were harvested and weighted, n = 6. The error bars represent s.d. values. The result was obtained without repetition.

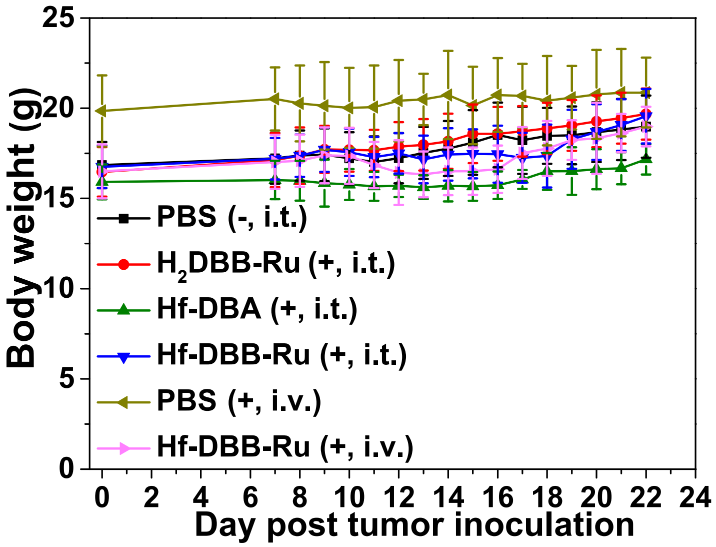

Supplementary Figure 25 Body weights curves of MC38 tumor model. MC38 tumor-bearing mice were treated with PBS, Hf-DBA, DBB-Ru, or Hf-DBB-Ru by intratumoral (i.t.) injection with (-) or without (+) X-ray irradiation or Hf-DBB-Ru by intravenous (i.v.) injection followed by X-ray irradiation and the body weights were monitored, n = 6. The error bars represent s.d. values. The result was obtained without repetition.

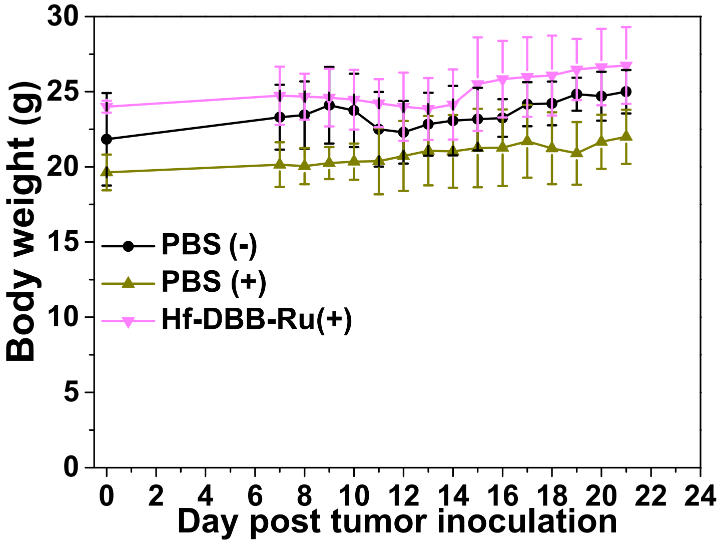

Supplementary Figure 26 Body weights curves of CT26 tumor model. Body weights were monitored after X-ray irradiation treatment on CT26 models treated with i.v. injected PBS or Hf-DBB-Ru, n = 6. The error bars represent s.d. values. The result was obtained without repetition.

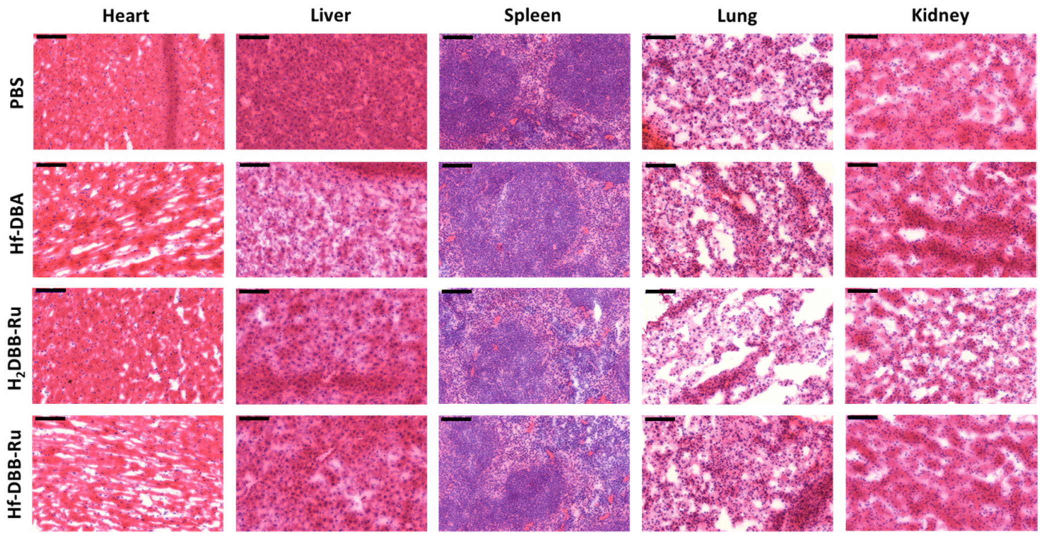

Supplementary Figure 27 Histologies of frozen sections of as treated mice after H&E staining. Major organs of MC38 tumor-bearing mice receiving intratumoral injection of Hf-DBA, Hf-DBB-Ru, H_2DBB-Ru or PBS and X-ray irradiation treatment were harvested and sectioned. From top to bottom: PBS, Hf-DBA, H_2DBB-Ru, and Hf-DBB-Ru, respectively. From left to right: heart, liver, spleen, lung or kidney. Scale bar = 100 μm. The result was obtained without repetition.

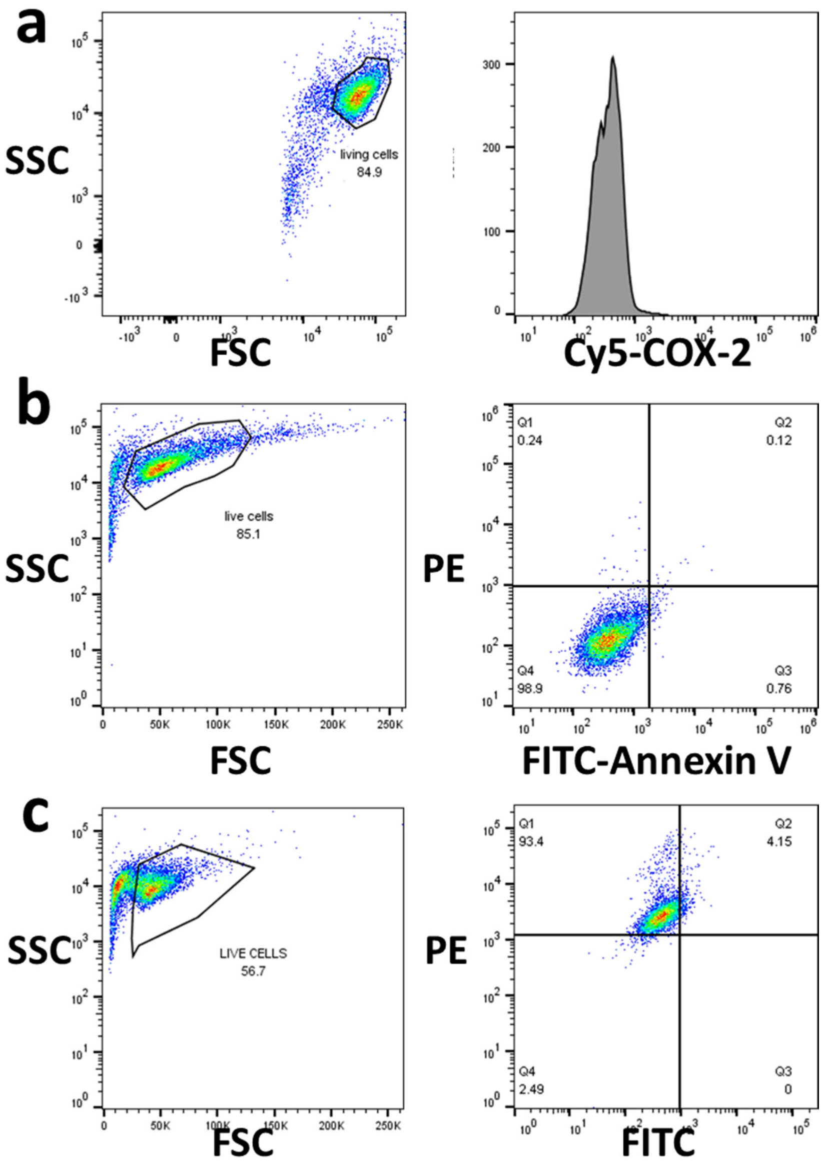

Supplementary Figure 28 Representative gating strategies for (a) COX-2 expression, (b) cell apoptosis, and (c) JC-1 analysis.

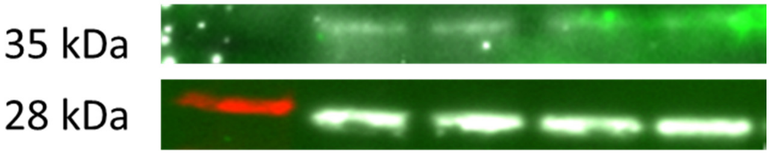

Supplementary Figure 29 Uncropped gel blots with markers. Upper: Caspase-3; Lower: Bcl-2.

Supplementary Table 1. EXAFS fitting parameters for H_2DBB-Ru

**Sample name**	H_2DBB-Ru
**Fitting range**	k: 2.87 – 12.11 Å^{-1}<br>R: 1.25 – 5 Å
**Independent points**	22
**Variables**	9
**Reduced chi-square**	443
**R-factor**	0.011
**S_0^2**	1.0
**ΔE_0(eV)**	-2.97
**R(Ru-N_5) (6)**	2.06 ± 0.01 Å
**σ^2(Ru-N_5)**	0.003 ± 0.002
**R(Ru-C_{15}) (6)**	2.91 ± 0.00 Å
**σ^2 (Ru-C_{15})**	0.002 ± 0.000
**R(Ru-C_{15}) (6)**	2.91 ± 0.00 Å
**σ^2 (Ru-C_{21})**	0.002 ± 0.000
**R(Ru-C_{15}) (6)**	4.25 ± 0.01 Å
**σ^2 (Ru-C_{14})**	0.01026 ± 0.000
**R(Ru-C_{15}) (6)**	4.83 ± 0.07 Å
**σ^2(Ru-C_{13})**	0.002 ± 0.002

Supplementary Table 2. EXAFS fitting parameters for Hf-DBB-Ru.

**Sample name**	Hf-DBB-Ru
**Fitting range**	k: 3.0 - 10.4 Å^{-1}<br>R: 1.2 - 5.5 Å
**Independent points**	20
**Variables**	7
**Reduced chi-square**	21.5
**R-factor**	0.015
**S_0^2**	1.0
**ΔE_0(eV)**	0.05
**R(Ru-N_5) (6)**	2.06 ± 0.01 Å
**σ^2(Ru-N_5)**	0.003 ± 0.000
**R(Ru-C_{15}) (6)**	2.91 ± 0.01 Å
**σ^2 (Ru-C_{15})**	0.002 ± 0.000
**R(Ru-C_{15}) (6)**	2.91 ± 0.01 Å
**σ^2 (Ru-C_{21})**	0.002 ± 0.000
**R(Ru-C_{15}) (6)**	2.91 ± 0.01 Å
**σ^2 (Ru-C_{14})**	0.016 ± 0.005
**R(Ru-C_{15}) (6)**	2.91 ± 0.01 Å
**σ^2(Ru-C_{13})**	0.002 ± 0.000

Supplementary Table 3. Statistical analysis of the tumor sizes at the end of treatment on CT26 or MC38 tumor bearing mice.

<table>
<tr><th rowspan="2"></th><th colspan="2">P values</th></tr>
<tr><th>CT26</th><th>MC38</th></tr>
<tr><td>PBS (+) vs PBS (-)</td><td>0.202</td><td>0.170</td></tr>
<tr><td>Hf-DBA (+, i.t.) vs PBS (+)</td><td rowspan="7"><0.0001</td><td>0.002</td></tr>
<tr><td>H_2DBB-Ru (+, i.t.) vs PBS (+)</td><td>0.024</td></tr>
<tr><td>Hf-DBB-Ru (+, i.t.) vs PBS (+)</td><td><0.0001</td></tr>
<tr><td>Hf-DBB-Ru (+, i.v.) vs PBS (+)</td><td><0.0001</td></tr>
<tr><td>Hf-DBB-Ru (+, i.t.) vs Hf-DBB-Ru (+, i.v.)</td><td>0.109</td></tr>
<tr><td>Hf-DBB-Ru (+, i.t.) vs Hf-DBA (+, i.t.)</td><td><0.0001</td></tr>
<tr><td>Hf-DBB-Ru (+, i.t.) vs H_2DBB-Ru (+, i.t.)</td><td><0.0001</td></tr>
</table>

Supplementary Table 4. Statistical analysis of excised tumor weights at the end of treatments on CT26 or MC38 tumor bearing mice.

	**P values**	
	CT26	MC38
PBS (+) vs PBS (-)	0.299	0.321
Hf-DBA (+, i.t.) vs PBS (+)		0.012
H_2DBB-Ru (+, i.t.) vs PBS (+)		0.025
Hf-DBB-Ru (+, i.t.) vs PBS (+)		0.003
Hf-DBB-Ru (+, i.v.) vs PBS (+)	<0.001	0.0015
Hf-DBB-Ru (+, i.t.) vs Hf-DBB-Ru (+, i.v.)		0.866
Hf-DBB-Ru (+, i.t.) vs Hf-DBA (+, i.t.)		0.0019
Hf-DBB-Ru (+, i.t.) vs H_2DBB-Ru (+, i.t.)		<0.001
